# Unexpected Inheritance: Multiple Integrations of Ancient Bornavirus and Ebolavirus/Marburgvirus Sequences in Vertebrate Genomes

**DOI:** 10.1371/journal.ppat.1001030

**Published:** 2010-07-29

**Authors:** Vladimir A. Belyi, Arnold J. Levine, Anna Marie Skalka

**Affiliations:** 1 Simons Center for Systems Biology, Institute for Advanced Study, Princeton, New Jersey, United States of America; 2 Institute for Cancer Research, Fox Chase Cancer Center, Philadelphia, Pennsylvania, United States of America; University of California Irvine, United States of America

## Abstract

Vertebrate genomes contain numerous copies of retroviral sequences, acquired over the course of evolution. Until recently they were thought to be the only type of RNA viruses to be so represented, because integration of a DNA copy of their genome is required for their replication. In this study, an extensive sequence comparison was conducted in which 5,666 viral genes from all known non-retroviral families with single-stranded RNA genomes were matched against the germline genomes of 48 vertebrate species, to determine if such viruses could also contribute to the vertebrate genetic heritage. In 19 of the tested vertebrate species, we discovered as many as 80 high-confidence examples of genomic DNA sequences that appear to be derived, as long ago as 40 million years, from ancestral members of 4 currently circulating virus families with single strand RNA genomes. Surprisingly, almost all of the sequences are related to only two families in the Order *Mononegavirales*: the Bornaviruses and the Filoviruses, which cause lethal neurological disease and hemorrhagic fevers, respectively. Based on signature landmarks some, and perhaps all, of the endogenous virus-like DNA sequences appear to be LINE element-facilitated integrations derived from viral mRNAs. The integrations represent genes that encode viral nucleocapsid, RNA-dependent-RNA-polymerase, matrix and, possibly, glycoproteins. Integrations are generally limited to one or very few copies of a related viral gene per species, suggesting that once the initial germline integration was obtained (or selected), later integrations failed or provided little advantage to the host. The conservation of relatively long open reading frames for several of the endogenous sequences, the virus-like protein regions represented, and a potential correlation between their presence and a species' resistance to the diseases caused by these pathogens, are consistent with the notion that their products provide some important biological advantage to the species. In addition, the viruses could also benefit, as some resistant species (e.g. bats) may serve as natural reservoirs for their persistence and transmission. Given the stringent limitations imposed in this informatics search, the examples described here should be considered a low estimate of the number of such integration events that have persisted over evolutionary time scales. Clearly, the sources of genetic information in vertebrate genomes are much more diverse than previously suspected.

## Author Summary

Vertebrate genomes contain numerous copies of retroviral sequences, acquired over the course of evolution. Until recently they were thought to be the only type of RNA viruses to be so represented. In this comprehensive study, we compared sequences representing all known non-retroviruses containing single stranded RNA genomes, with the genomes of 48 vertebrate species. We discovered that as long ago as 40 million years, almost half of these species acquired sequences related to the genes of certain of these RNA viruses. Surprisingly, almost all of the nearly 80 integrations identified are related to only two viral families, the Ebola/ Marburgviruses, and Bornaviruses, which are deadly pathogens that cause lethal hemorrhagic fevers and neurological disease, respectively. The conservation and expression of some of these endogenous sequences, and a potential correlation between their presence and a species' resistance to the diseases caused by the related viruses, suggest that they may afford an important selective advantage in these vertebrate populations. The related viruses could also benefit, as some resistant species may provide natural reservoirs for their persistence and transmission. This first comprehensive study of its kind demonstrates that the sources of genetic inheritance in vertebrate genomes are considerably more diverse than previously appreciated.

## Introduction

The integration of a DNA copy of the retroviral RNA genome into the DNA of infected cells is an essential step in the replication of these viruses. Portions of DNA tumor virus genomes can also become integrated into cellular DNA, but this is a relatively rare event, detected by selection of a clone of cells that express the viral oncogene(s). While such integration events occur routinely in somatic cells, retroviral DNA sequences are also integrated in the germlines of many hosts, giving rise to inherited, endogenous proviruses. It has been reported that sequences from viruses that contain RNA genomes and do not replicate through a DNA intermediate, may also be copied into DNA and become integrated into the germline cells of plants and insects [1], [2], [3]. That such events can have biological impact was demonstrated in the case of sequences derived from the positive strand RNA genome of a Dicistrovirus (Israeli acute paralysis virus), which were integrated into the germline of bees from different hives [2]. Bees with genomes that contain sequences encoding a portion of the structural protein of this virus are resistant to infection by this same virus. Similar observations have been made in mice with endogenous retroviral sequences related to a capsid gene (*Fv-1* locus) which confers resistance to infection by some retroviruses [4]. These observations suggest that chronic infections of a host with both retroviruses and non-retro RNA viruses can result in germline integration events that produce a host expressing some viral functions that confer an advantage to the species; resistance to subsequent infection by that virus.

With these ideas in mind, we undertook a search in the germline genomes of vertebrates for DNA sequences that may be related to any of the known non-retroviral families of viruses that contain single-stranded RNA genomes. As our analyses were being completed, an independent group of investigators reported that sequences derived from the nucleocapsid gene (N) of ancient relatives of such a virus, the Borna disease virus (BDV), are integrated in the genomes of several mammalian species [5]. Here we report the results of our comprehensive search in which 5,666 sequences from non-retroviruses with RNA genomes were compared with the DNA sequences in the genomes of 48 vertebrate species. Our studies have not only confirmed the integration of BDV N-related sequences, but they have also revealed that sequences related to the matrix and polymerase genes of this virus have been integrated into the germlines of various vertebrate species. In addition, we have discovered genome integrations of viral gene sequences from other members of the order *Mononegavirales*, with the most prominent related to Ebolaviruses and Lake Victoria Marburgvirus. It is noteworthy that these viruses exhibit extremely high mortality rates in some susceptible species, for example reaching 80% in horses that develop Borna disease, and up to 90% in humans infected with Ebolavirus [6].

**Figure 1:**
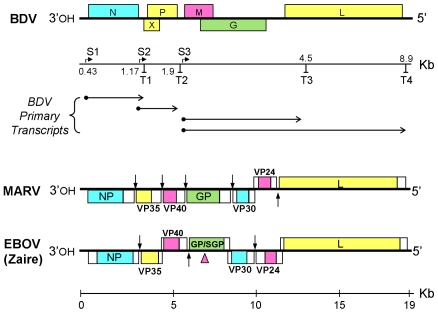
Organization and transcription maps of Borna disease virus (BDV), Marburgvirus (MARV) and Ebolavirus (EBOV) genomes. Open reading frames are labeled and indicated by colored boxes, non-coding regions by empty boxes. For BDV, the locations of transcription initiation (S) and termination (T) sites are shown on the scale beneath the genome map. The horizontal arrows below the scale depict the origins of primary transcripts. The two longest BDV transcripts are subjected to alternative splicing to form multiple mature mRNAs. For MARV and EBOV, vertical arrows indicate transcription initiation and termination sites, except for regions of overlap, where these sites are not marked. The pink arrowhead points to the location of an editing site in the GP gene of EBOV.

In addition to possessing linear non-segmented, negative sense single-stranded RNA genomes, the *Mononegavirales* have several other features in common, including a similar gene order and transcription strategy in which genes are flanked by specific transcription start and stop sites and are expressed in a gradient of decreasing abundance (Figure 1, for review see: [7]). The 8.9 Kb BDV genome encodes information for at least six proteins. These viruses form a unique family, the *Bornaviridae*, and they are the only viruses in the Order to replicate and transcribe their genomes within the nucleus of the infected cell [8]. Sheep, horses, and cows are among the natural hosts for this enzootic virus; while there are a number of other experimental hosts, virus replication under such conditions is poor, chronic, and slow [8]. Many tissues can be infected in susceptible hosts, but disease symptoms are commonly neurological. Natural infections of humans are at best controversial, and infectious virus has been isolated from this source only infrequently [9]. Given that the BDV is an RNA virus, its genome sequence conservation among isolates of many mammalian species, separated in both time and geographic locations, is surprisingly high. This suggests strong selection pressure to retain a core sequence for virus viability in a reservoir species with which an evolutionary equilibrium has been established.

The Ebola (EBOV)- and Marburg (MARV)- viruses comprise the two genera of the family *Filoviridae*. Their approximately 19 Kb genomes are replicated and transcribed in the cytoplasm of infected cells. EBOV and MARV cause highly lethal hemorrhagic fever in humans and have high potential for individual-to-individual transmission. Several strains of EBOV are known, including the Zaire and Sudan strains in Africa, and the Reston strain in the Philippines. The latter has only been associated with monkeys, but a recent report also found infection by this strain in domestic swine, and the presence of antibodies in six exposed farm workers [10]. Recent evidence suggests that bats are the natural reservoir of these zoonotic agents [[11], and references therein,[12]].

## Results

### Distribution of RNA virus-like sequences among vertebrate species

To conduct this survey, a BLAST program (see Methods) and the NCBI viral Refseq database of virus sequences were employed (October 2009 release) which, at the time, contained a total of 79,001 viral protein sequences, among them 5,666 sequences from viruses with single-stranded RNA genomes that replicate without a DNA intermediate. The latter sequences included all 4 known Orders of animal viruses?with single-stranded RNA genomes, and represented all 38 recognized families, as well as 9 additional unclassified viral genera with such genomes. These viral sequences were compared with 48 complete vertebrate genomes, to determine if any could be identified in the vertebrate genomes. The results were striking, revealing numerous genomic sequences related primarily to two currently circulating virus families with single, negative strand RNA genomes, the Bornaviruses and Filoviruses (Table 1). Selected examples are listed in Table 2, with a complete list provided in Supporting Tables S1, S2, S3, S4, S5, S6 and S7 and Figures S3 and S4. The most numerous of these virus-like sequences were related to the nucleocapsid N (p40) gene of BDV, but sequences related to the BDV RNA-directed RNA polymerase (L), and to the genes encoding the major nucleocapsid protein, NP, and the minor nucleocapsid, polymerase complex cofactor protein (VP35) of EBOV/MARV, were also detected in several vertebrate genomes. Sequences related to the matrix protein (M) gene of BDV were detected in the lemur and medaka genomes, and to the L gene of EBOV/MARV in the opossum genome. Altogether, we discovered BDV-like sequences in at least 13 species, and EBOV/MARV-like sequences in at least 6 species. A single, high confidence example of sequences related to the L gene of Midway/Nyamanini virus was detected in the zebrafish genome. A sequence related to the Tamara Bat virus in the medaka genome was the lone example related to a positive strand RNA viral genome. In many of these examples no synteny was observed among chromosome locations of the sites in different related vertebrate genomes and we conclude that most represent independent integration events, possibly taking place over extended time periods. In other cases, both synteny of chromosomal locations and copy number stability in a genome is observed for virus-related sequences, through lines of inheritance.

**Table 1:** Sequences derived from single strand RNA viral genes, which are integrated in mammalian genomes.1)

	Borna Disease Virus			Filoviruses2) (Ebolavirus, Marburgvirus)			Midway (or similar) Virus	Tamana Bat Virus (or other Flaviridae)
	N	M	L	NP	L	VP35	L	NS3
Primates	+							
Bushbaby	+							
Lemur	+	+						
Tarsier	+					+		
Mouse	+		+					
Rat	+		+					
Squirrel	+							
Guinea pig	+			+/−				
Cow	+/−							
Microbat	+		+	+		+		
Shrew				+/−				
Opossum	+			+	+			
Wallaby	+/−		+	+		+		
Medaka		+/−	+					+/−
Takifugu			+					
Zebrafish							+	
Lamprey	+							

1) Integrations with BLAST E-value below 10^−10^ are labeled with plus sign “+”. Integrations with E-value as high as 10^−5^ are marked “+/−”: these may be derived from earlier infections or infections with a different strain of the virus. All integrations were cross checked against the NCBI database of protein and nucleotide sequences to confirm the viral origin of the sequence. Species are listed in the reverse chronological order from the time they shared common ancestor with humans.

2) While Ebolavirus and Marburgvirus are now recognized as different virus genera, their sequences are closely related. Accordingly, it is not possible to uniquely associate integrated fragments with either virus.

**Table 2:** Selected endogenous viral sequences found in vertebrate genomes.

Specie	Scaffold or Chromosome	Virus	Integrated gene	Location within present-day virus protein	Viral protein length	BLAST hit, E-value and percent identity1)	Label	Significant large ORFs (length and position)
Human (*Homo Sapiens*)	chr10	Bornavirus	N	28–349	370aa	2E-65/41%	hsEBLN-1	366aa (full protein)
Squirrel (*Spermophilus Tridecemlineatus*)	scaffold113120	Bornavirus	N	40–368	370aa	1E-155/77%	stEBLN	203aa (residues 170–370)
Microbat (*Myotis Lucifugus*)	scaffold144630	Reston Ebolavirus	VP35	74–329	329aa	5E-23/30%	mlEEL35	281aa (residues 52–329)
Tarsier (*Tarsius Syrichta*)	scaffold521	Reston Ebolavirus	VP35	138–329	329aa	5E-16/34%	tsEEL35	131aa (residues 137–261)
Grey Mouse Lemur (*Microcebus Murinus*)	scaffold5488	Bornavirus	M	1–123	142aa	4E-13/45%	mmEBLM	93aa (residues TSS-102)
Medaka *(Oryzias Latipes*)	scaffold1213	Bornavirus	M	15–138	142aa	5E-07/33%	olEBLM	69aa (residues TSS-71)
Microbat (*Myotis Lucifugus*)	scaffold114379	Bornavirus	L	189–1066	1608aa	3E-96/42%	mlEBLL-1B	149aa
Microbat (*Myotis Lucifugus*)	scaffold131047	Lake Victoria Marburgvirus	N	63–437	695aa	2E-36/32%	mlEELN-1	158aa (residues 72–228) and 164aa (residues 228–391)
Opossum (Monodelphis Domestica)	chr2	Reston Ebolavirus	NP	175–409	739aa	4E-39/46%	mdEELN	no significant ORF found
Wallaby (*Macropus Eugenii*)	scaffold117569	Sudan Ebolavirus	NP	22–312	738aa	1E-28/33%	meEELN-5	>218aa likely (incomplete scaffold)
Opossum (Monodelphis Domestica)	chr3	Lake Victoria Marburgvirus	L	605–1354	2331aa	5E-72/	mdEELL	no significant ORF found
Zebrafish (*Danio Rerio*)	chr25	Midway Virus	L	238–962	1935aa	8E-027/21%	drEMLL-3	761aa (residues TSS-756)
								180aa (residues 792–971)

1) Only the top BLAST E-value and average percent identity are shown when BLAST alignment returns multiple gene fragments. Please refer to supplementary data (Tables S1, S2, S3, S4, S5, S6 and S7) for a complete list of integrations and individual BLAST hits.

### How did the endogenous RNA virus-likes sequences become incorporated into the genomes of their hosts?

The genes of viruses in the Order *Mononegavirales* are transcribed as mono- or dicistronic mRNAs (Figure 1). The distribution of endogenous virus-like sequences that were detected here, appear to be limited to one or very few per specie. This, and the fact that single genes are represented in diverse locations, is suggestive of a mechanism that involved the reverse transcription and integration of DNA copies of viral mRNAs by LINE elements, much as cellular pseudogenes are produced. Indeed, we found several cases in which landmarks, or remnants of landmarks, characteristic of Line element-mediated insertion are associated with specific Bornavirus- and Filovirus-related integrations. These include direct repeats flanking transcription start sites and 3′ polyA sequences (Table 3). In many additional cases, only 3′ polyA sequences were observed (data not shown). The fact that direct repeats are not found for some endogenous sequences is not surprising, as these repeats may be just 2 nucleotides long and likely have experienced numerous mutations from the time of initial integration. However, from the informative examples in Table 3 we conclude that some, if not all, RNA virus-related sequences have been integrated into their host genomes by LINE elements via target-primed reverse transcription from ancient viral mRNAs.

**Table 3:** Presence of direct repeats, viral transcription start sites, and poly-A sequences in some virus-related genomic integrations.

Insertion	Direct repeat and 5′ TSS sequence	TSS location1)	3′ Poly-A sequence and direct repeat	Poly-A location2)
**Bornavirus-related endogenous sequences**				
Human hsEBLN-2	AG**AATTAAGTC**GGAACCAATTTTCCACAATGT…	−3	… TTAAAAAAA …AluSx3)… TAAAAAAA **AATTAAGTC**A	1107
Human hsEBLN-3	TA**GATCTGGGCATAG** GAACCAATCAGAAACAATCG…	−10	… TTAAAAAAAAAAAAA **GATTTGGGCATAG**ATTG	1133
Human hsEBLN-4	TAAGACAAC**AAAG** GAACCGATTGCTCCCGCAGC…	−3	… TTAAAAAAAAA **AAAG**CCGCTCCTCAGACC	1133
Human hsEBLN-1	AT**TGTGTGAAAATCACA** GAAACAATCACCCACAATGTC…	−21	… TATAAAAAGAAATTA **TGTGAAAATCACA**TTCTAA	1126
**Filovirus-related endogenous sequences**				
Microbat mlEELN-2	GAC**AGTATTTC**AGAGGAACATTAG …	+21	… TTAAGAAAAAAAAAGTAAAT**AGTATTTC**TG	2212
Microbat mlEEL35	TCTCCCACCTCA**AAGA**TGAGAGGATTTTTAA …	−65	… TTAAGAAAAAG**AAGA**AAAAAGAATAGGACAA	1869
Tarsier tsEEL35	CTAAATAAATAG**TGTG**GGAGGAACATTAA …	−134	…GTAAGAAAAAAT**TGTG**AGTTAAATTTATTT	1311

Sequences most resembling the canonical transcription start site (TSS) and canonical poly-A sequences are underlined. Direct repeat sequences flanking virus-derived integrations are shown in bold.

1) Location of the TSS relative to the estimated position of the coding sequence start, based on the present day viral protein. The expected location is −11 for Bornavirus EBLN insertions, −414 for EBOV EELN insertions, −55 for MARV EELN insertions; and −92 to −97 for EEL35 insertions.

2) Location of the poly-A sequence relative to the estimated position of the coding sequence start, based on the present day viral protein. The expected location is 1110 for Bornavirus EBLN insertions, 2545 for EBOV EELN insertions, 2730 for MARV EELN insertions, and 1268 to 1455 for EEL35 insertions.

3) Bornavirus integration hsEBLN-2 in human genome is directly followed by a repeat element AluSx, also observed by Hoire et al [5]. These two integrated sequences are surrounded by a common direct repeat.

### When were the RNA virus-like sequences integrated?

**Figure 2:**
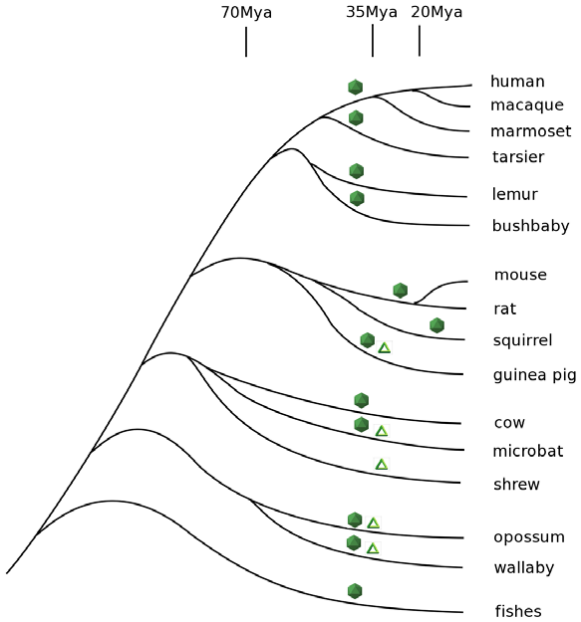
Phylogenetic tree of vertebrates that encode Bornavirus- and Filovirus- like proteins in their genomes. Bornaviruses-related sequences are denoted by icosahedrons and Filoviruses-related sequences by triangles. Times of the viral gene integrations are approximate, unless discussed in the text.

In some cases, the integrations of virus-related genes were observed in closely related species descended from each other, allowing an estimate of the oldest common ancestor of these integrations. For example, a rodent lineage (including mice and rats) contains BDV gene N- and L-related endogenous sequences, and a separately derived primate lineage (comprising marmosets, macaques, chimps, and humans) contains endogenous BDV gene N-related sequences integrated into seven different places in the genomes. The rodent and primate lines differ from each other in their integration sites, but within both lineages identical sites of integration and stable copy numbers of genes are observed, indicating decent through lineages of viral genes integrated in the past. In the primate line these sites first appear in the present day marmosets and have been retained over forty million years from a common ancestor of marmosets and humans (Figure 2). Based on the degree of sequence homology of BDV-related genes in different host genomes, most of these integrations seem likely to have originated in the same time frame, with the exception of the integration in squirrels, which has much higher sequence homology to the present day virus (Table S1). We stress that integration events illustrated in Figure 2 appear to have been independent events, and do not come from a single ancient integration: no synteny in integrated sequences and adjacent chromosome is observed across species.

**Figure 3:**
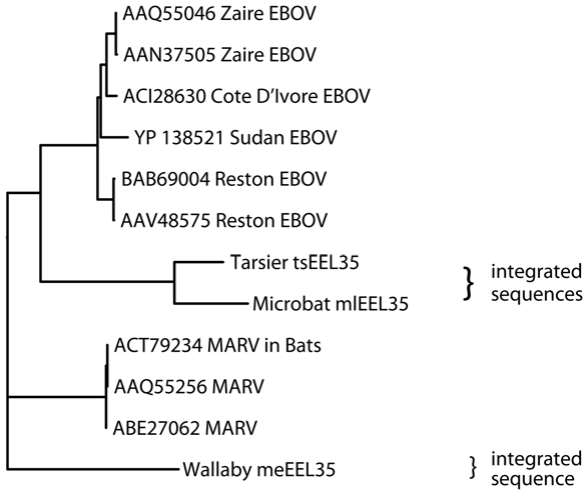
Phylogeny of endogenous Filovirus VP35 - like gene integrations. The tree was built with PHYLIP based on ClustalW alignment using only aligned residues present in all sequences. The tree is unrooted (the wallaby integration was used as an outgroup for given representation). Bootstrap values are at least 92, with the exception for Sudan EBOV (54), Cote D'Ivore EBOV (77), and MARV in bats (70).

The timing of integrations of the EBOV/MARV-related sequences is less clear. The examples of these viral gene sequences fail to distinguish between the present day strains of EBOV and Lake Victoria MARV suggesting an ancient ancestor of both (Figure 3 and Figure S1). Because the integration events appear to predate the split between these genera, we consider them together, and have estimated their ages indirectly. We start with the assumption that at the time of integration, functional protein-coding sequences were free of stop codons. Some of these integrated viral sequences appear to be under positive selection to the present day, because they have retained their open reading frames. Other integrated viral gene sequences have not retained open reading frames and have mutation rates that are measurable. We can employ the latter to estimate the age of an integration event. The typical rate of vertebrate genetic drift ranges from 0.12% of nucleotides per million years in primates to 2–4 times that value in rodents [13], [14], [15], [16]. There are three stop codons and nineteen codons that can become stop codons with a single base change. Assuming an equal frequency of all possible single nucleotide changes, there is a 12% probability that a random codon change will produce a stop codon in one mutational step. Genomic sequences that once encoded proteins, but are now non-functional pseudogenes, are therefore expected to develop stop codons at a rate of one per 1/(0.12×3×0.0012)≈2310 positions for each million years of evolution of primates, and 2–4 times more frequently in rodents.

We next analyzed virus-derived integrations for the presence of stop codons in the stretches of aligned peptide sequences, as shown in Table 4 (additional integrations are listed in Table S8). According to the calculations described above, the two least conserved, near full-length integrations of BDV-related genes in humans, hsEBLN-3 and hsEBLN-4, appear to be 48 and 40 million years old respectively, consistent with our earlier estimates based on primate phylogeny. Integrations in rodents appear to be more recent, or have lost their protein coding ability at a later time, about 21 million years ago for rodEBLL and 19 million years for rodEBLN-2 and rodEBLN-4. Interestingly, the mouse integrations appear to be under stronger selection that those in rats. The EBOV/MARV-related integrations in the opossum genome appear to be 32–53 million years old (assuming 0.13% neutral rate for nucleotide drift per million years [17]). The ages cited here are rough estimates, as rates of genetic drift vary in time and across different stretches of DNA. Other integrations have similar sequence identity with the present day viruses and appear to originate from the same time in history. However, we do not explicitly cite their ages due to the preliminary nature of the scaffold assemblies for carrier species (Table 4 and Table S8).

**Table 4:** List of representative vertebrate integrations found by BLAST search and total number of stop codons inside aligned peptide regions.1)

Integration	Specie	Virus	Integrated gene	Total number of stop codons	Total length of BLAST alignments	Sequence identity	Number of stop codons per 100 aminoacids
drEMLL-4	Zebrafish	Midway Virus	L	0	365	22%	0.0
stEBLN	Squirrel	Bornavirus	N	0	329	77%	0.0
hsEBLN-1	Human	Bornavirus	N	0	318	41%	0.0
mlEEL35	Microbat	Ebola/Marburgvirus	VP35	0	263	30%	0.0
laEBLN-2	Elephant	Bornavirus	N	0	256	32%	0.0
mlEBLL-2B	Microbat	Bornavirus	L	0	229	37%	0.0
tsEEL35	Tarsier	Ebola/Marburgvirus	VP35	0	191	34%	0.0
olENS3	Medaka	Tamana Bat Virus	NS3	0	190	28%	0.0
ogEBLN-1	Bushbaby	Bornavirus	N	0	168	29%	0.0
mlEBLN-1	Microbat	Bornavirus	N	0	168	29%	0.0
saEBLN-1	Shrew	Bornavirus	N	0	167	31%	0.0
ogEBLN-3	Bushbaby	Bornavirus	N	0	138	34%	0.0
drEMLL-3	Zebrafish	Midway Virus	L	1	712	21%	0.1
rodEBLN-4	Mouse	Bornavirus	N	1	269	40%	0.4
rodEBLN-3	Rat	Bornavirus	N	1	263	38%	0.4
mlEELN-3	Microbat	Ebola/Marburgvirus	NP	1	204	42%	0.5
rodEBLN-3	Mouse	Bornavirus	N	2	314	37%	0.6
trEBLL	Fugu	Bornavirus	L	3	458	43%	0.7
olEBLL	Medaka	Bornavirus	L	3	340	44%	0.9
mmEBLM	Lemur	Bornavirus	M	1	112	45%	0.9
cpEBLN	Guinea Pig	Bornavirus	N	2	207	41%	1.0
hsEBLN-2	Human	Bornavirus	N	3	309	38%	1.0
rodEBLN-1	Rat	Bornavirus	N	3	289	43%	1.0
meEELN-5	Wallaby	Ebola/Marburgvirus	NP	3	280	33%	1.1
meEBLL-1	Wallaby	Bornavirus	L	4	364	39%	1.1
rodEBLN-2	Mouse	Bornavirus	N	3	266	39%	1.1
saEBLN-2	Shrew	Bornavirus	N	2	152	31%	1.3
mdEELL	Opossum	Ebola/Marburgvirus	L	8	520	31%	1.5
tsEBLN	Tarsier	Bornavirus	N	2	130	37%	1.5
hsEBLN-4	Human	Bornavirus	p40	4	234	37%	1.7
olEBLM	Medaka	Bornavirus	M	2	114	33%	1.8
mimEBLN	Lemur	Bornavirus	N	5	277	35%	1.8
rodEBLL	Rat	Bornavirus	L	12	640	35%	1.9
meEEL35	Wallaby	Ebola/Marburgvirus	VP35	4	201	31%	2.0
hsEBLN-3	Human	Bornavirus	N	6	288	43%	2.1
meEELN-4	Wallaby	Ebola/Marburgvirus	NP	6	272	41%	2.2
rodEBLL	Mouse	Bornavirus	L	16	693	28%	2.3
mdEBLN-1	Opossum	Bornavirus	N	7	302	32%	2.3
laEBLN-3	Elephant	Bornavirus	N	7	297	33%	2.4
mdEELN	Opossum	Ebola/Marburgvirus	NP	6	237	46%	2.5
btEBLN	Cow	Bornavirus	N	5	192	28%	2.6
mlEELN-2	Microbat	Ebola/Marburgvirus	NP	11	419	44%	2.6
meEELN-12	Wallaby	Ebola/Marburgvirus	NP	8	262	39%	3.1

1) Only representative hits per specie are listed. Additional integrations are shown in Supplementary Table S8.

### Preservation of open reading frames

The absence of the stop codons in some integrations points to strong selective pressures towards maintenance of full-length open reading frames. This is in contrast to the actual peptide sequences that appear to be undergoing neutral drift. Over the 20 million years of evolution in rodents and 40 million years in other mammals, we expect a 5–10% nucleotide change or approximately 15–30% codon change, if there is no selective pressure against fixation of such events in the population. Accordingly, one would expect to observe a stop codon in 1.8–3.6% of the codons. This is, indeed, the case for the majority of the integrations (Table 4 and Table S8). In contrast, several integrations show signs of strong positive selection, namely those related to the BDV N gene in humans, microbats, rodents, and other animals, and both the EBOV/MARV NP and VP35 gene-related integrations in bats and tarsier. Some integration events, including the BDV N-like sequences in humans (e.g. hsEBLN-1) and the EBOV VP35-like sequences in microbats (mlEEL35) have maintained nearly full-length open reading frames (Table 2). The probability of having no stop codon in the longest of these, the BDV gene N-like integration in humans, is one in eight hundred, suggesting that at some time, past or present, there was strong selective pressure to keep and express this ancestral viral gene.

### Are some endogenous RNA virus-like sequences expressed?

Expressed sequence tags (EST) were identified for four integrated copies of the BDV N-related genes in humans (hsEBLN-1 through hsEBLN-4). The chromosome 3 integration (hsEBLN-2) is actually tiled on Affymatrix chips to detect mRNAs from human tissues. Analysis of a very large diversity of tissue types show low levels of this transcript in most tissues tested, intermediate levels in thymus, olfactory bulb, fetal thyroid, liver, prefrontal cortex, CD34 cells, endothelial cells and dendritic cells, and high levels in CD4 and CD8 T-cells (Figure S2). In susceptible species, BDV replicates mainly in cells of the nervous system, but viral nucleic acids and proteins have been isolated from peripheral blood mononuclear cells. It is clear that several BDV N-like endogenous sequences are expressed as mRNAs in human tissues. Expression of mRNA from these endogenous sequences was also detected in several cell lines in cell culture [5].

### Is the expression of endogenous RNA virus-like sequences biologically relevant?

BDV is an enzootic virus, with natural infections occurring in sheep, horses, and cattle [18], in which serious, often fatal, neurological symptoms are observed. These animals have no detectable copies of the BDV-related endogenous sequences. Furthermore, species in the primate and mouse/rat lineages, which contain endogenous N-like sequences, are generally resistant to the virus, or the virus is observed to replicate poorly with little or no symptoms in these animals [19] (Table 5). In cows, which do have endogenous sequences related to the BDV N gene, there is apparently no present day selection for its coding capacity (Table 4), and cows are known to be susceptible to Borna disease. Thus, there appears to be a general correlation between natural resistance to the pathogenic effects of the virus and the potential for expression of BDV N-like endogenous sequences in a host. However, as has been observed with *Fv-1* in mice [20], natural resistance can be overcome under experimental conditions in which animals or cell cultures may be subjected to large doses of the virus (Table 5).

**Table 5:** Borna disease virus integrations and known host susceptibility to Borna disease.

Host	Known gene integrations	Natural viral host	Experimental infection
Primates/humans	N	No	Yes
Rodents (mice, rats)	N, L	No	Yes
Lemur	N, M		
Tarsier	N		
Dogs	-	Yes	Yes
Horses	-	Yes	
Cows	N (?)	Yes	Yes
Rabbits	-	Yes	
Donkeys	not sequenced1)	Yes	
Sloth	-	Yes	
Sheep	not sequenced1)	Yes	
Pigs	-		
Birds	-	Yes	Yes
Opossum, wallaby	N, L		
Guinea pig	N		Yes
Squirrel	N		

1) Whole genome sequences of donkey and sheep were not available at the time of writing.

**Figure 4:**
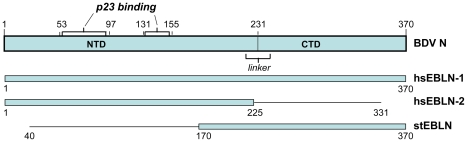
Domain structure of BDV N (p40) protein, and its alignment with open reading frames encoded in human and squirrel endogenous BDV N-like sequences. Shaded blue rectangles show open reading frames as seen in today's integrations. Solid black lines show total alignment found by BLAST.

The X-ray crystal structure of the N protein of BDV has been solved, and a number of critical features determined [21]. The protein is organized in two domains, separated by a short linker, and assembles into a homotetramer. We find that open reading frames in two endogenous human inserts, hsEBLN-1 and hsEBLN-2, are long enough to encode folded N-terminal domains (Figure 4), while an open reading frame in hsEBLN-1 also encodes a complete C-terminal domain. When expressed, either of these proteins could conceivably affect the proper assembly of the BDV ribonucleoprotein complex. Production of N-related antibodies might also inhibit virus replication. An open reading frame is also observed in the integration in squirrels (stEBLN), encompassing a complete C-terminal domain. The BDV gene N-related integration in the genome of the microbat *Myotis lucifugus* (mlEBLN-1), might also be found to carry a full-protein open reading frame when the preliminary assembly of this genome undergoes final revision.

EBOV and MARV are zootropic viruses that cause infections with some of the highest mortality rates in humans, primates, and pigs. Recent studies have suggested that megabats, specifically *Hypsignathus monstrosus*, *Epomops franqueti*, and *Myonycteris torquata*, could be potential natural reservoirs for EBOV [22]. Later studies also identified microbat *Mops condylurus*, as well as several other megabats, as potential reservoirs [11]. Some of the bats actually carry live virus, yet exhibit no visible symptoms of disease. There are more than 1,100 recognized species of bats, comprising about a fifth of all mammalian species [23], but the genomes of only two bat species have been sequenced. Our results show that at least one of them, the microbat *Myiotis lucifugus*, has detectable integrations of EBOV/MARV-like sequences, with several of these showing strong selective pressure for maintaining open reading frames (Table 4).

**Figure 5:**
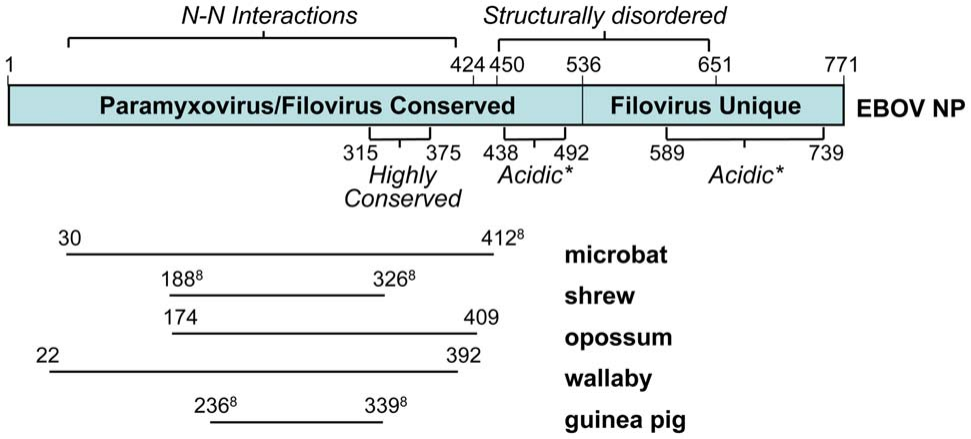
Domain structure of the EBOV N protein, and its alignment with several related endogenous sequences identified by the BLAST program. Amino acid coordinates marked with (&) have been mapped to the Zaire strain of Ebolavirus and may differ slightly from coordinates in Supplemental Table S4.

The most widespread EBOV/MARV integrations observed in this study are derived from the major viral nucleocapsid gene NP and the minor nucleocapsid and polymerase complex cofactor gene VP35. The endogenous sequences related to the NP protein align with the amino-terminal region (Figure 5), which is conserved among these viruses and the Paramyxovirus family, and is critical for NP-NP protein interactions [24], [25]. The microbat sequence mlEELN-1, for example, covers most of this region, including a highly conserved stretch of amino acids and part of a structurally disordered acidic region, which is thought to play a role in the incorporation of the protein into virus particles [24].

**Figure 6:**
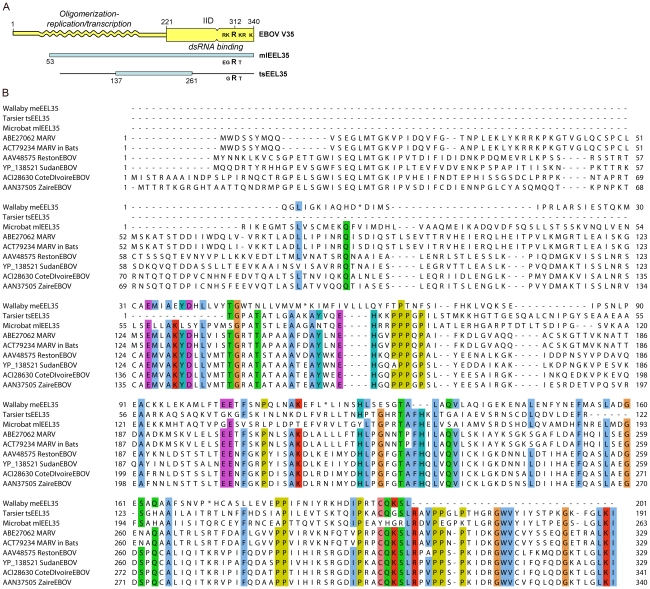
Comparisons of Filovirus VP35 protein sequences with those of related endogenous sequences. A) Domain structure of the EBOV (Zaire) VP35 protein, and its alignment with related endogenous sequences in the microbat and tarsier genomes. Shaded blue rectangles show open reading frames as seen in today's integrations. Solid black lines show total alignment found by BLAST; B) multiple alignment of endogenous sequences in wallaby, tarsier, and microbat, with the present day strains of EBOV and MARV. We used the default color scheme for ClustalW alignment in the Jalview program.

Determination of the X-ray crystal structure of the interferon inhibitory domain (IID) of the EBOV VP35 protein has identified two interacting sub-domains, the C-terminal of these includes a cluster of basic residues, centering on R312, which are critical for RNA binding [26]. The microbat endogenous sequence mlEEL35 encompasses the entire IID domain as well as a good portion of the N-terminal domain, which is required for VP35 oligomerization as well as viral replication and transcription (Figure 6). A comparison of the sequences shows that residues important for interactions between the IID sub-domains are largely conserved in mlEEL35 [27], [28]. However, while an arginine residue corresponding to R312 is retained in microbats and the tarsier, two or more of the surrounding acidic residues are substituted in each of these endogenous sequences. Substitution of these residues in EBOV VP35 diminishes RNA binding and abrogates the interferon antagonist function of this protein [26], [27]. Furthermore, viruses that carry these relevant mutations are non-pathogenic in normally susceptible guinea pigs, and animals infected with this mutated virus develop antibodies that render them resistant to subsequent challenge [29].

### Sequences in the vertebrate genome that are related to RNA virus glycoproteins

Our sequence search also uncovered what appear to be remnants of ancient integrations of virus-like glycoprotein genes (G), which are most similar to the glycoproteins from the Order *Mononegavirales* (Table 6). A BDV gene G-like integration in primates was acquired sometime before the split between humans and old world monkeys, and there are several integrations that most resemble the Filovirus glycoprotein genes (GP). In the Filoviruses, the GP precursor protein is cleaved to form two bound peptides, GP1 and GP2. We found no traces of receptor-binding GP1 [30] in the vertebrate genomes analyzed. However, we identified several sequences related to the second peptide, GP2, which is involved in glycoprotein trimerization [31], and is highly conserved among known Filoviruses (Table 6). Because GP2 shares sequence elements with the avian sarcoma/leukosis virus, the flanking regions of the top BLAST glycoprotein hits were checked for retroviral sequences, LTR elements and *gag*-*pol* genes (as described in Methods), and integrations that show no known adjacent retroviral elements were identified. Nevertheless, some ambiguity remains due to the preliminary nature of several of the vertebrate genome assemblies.

**Table 6:** Glycoprotein integrations sites.

Specie	Total Number of BLAST hits	Hits that have no retroviral *gag*, *pol*, and LTR elements1)	Virus2)	Glycoprotein residues	BLAST E-value and percent identity
Human	1	chr1: 46259885–46260178	Bornavirus	32–126	1E-07/37%
Chimp	1	chr1: 46259885–46260178	Bornavirus	32–126	6E-08/38%
Baboon	1	on several partial scaffolds	Bornavirus	6–155	3E-13/38%
Gorilla	1	gene scaffold 2544: 11117–11408	Bornavirus	59–155	6E-11/43%
Macaque	1	chr1: 48,422,406–48,422,783	Bornavirus	6–126	1E-10/35%
Tarsier	7	scaffold 99624:1,564–1,884	Reston Ebolavirus	497–610	2E-11/30%
Kangaroo rat	1	scaffold 40120: 1783–2128	Marburgvirus	509–628	4E-08/40%
Stickleback	1	chrVIII: 8,031,996–8,032,262	Reston Ebolavirus	533–628	2E-07/32%
Shrew	4	scaffold 231484:15,984–16,259	Reston Ebolavirus	559–648	3E-06/29%
Horse	13	chr10: 13,359,493–13,359,900	Reston Ebolavirus	508–652	3E-06/27%
Zebrafish	10	chr15: 6,268,907–6,269,284	Sudan Ebolavirus	516–651	3E-05/27%
		zv8_NA3400: 7,797–8.099	Reston Ebolavirus	519–628	2E-07/32%
Tetraodon	1	chr1:15,715,939–15,716,202	Zaire Ebolavirus	532–626	1E-06/29%
Fugu	1	chrUn:120,943,623–120,943,895	Zaire Ebolavirus	530–627	1E-07/31%
Sloth	4	None			
Cow	1	None			
Squirrel	1	None			
Platypus	3	None			
Chicken	8	None			
Zebrafinch	21	None			

1) All regions were tested for nearby *gag*, *pol*, and LTR elements to eliminate sequences of retroviral origin, as described in the methods section.

2) Only the most similar strain of virus is shown for filovirus-like integrations.

Assuming that the endogenous glycoprotein encoding sequences are, indeed, related to viruses in the Order *Mononegavirales*, their integration may also play role in virus resistance. For example, expression of a GP2 peptide from endogenous sequences may affect the trimerization of GP from a related infecting virus. Recent studies have indicated that over-expression of Filovirus GP in host cells may prevent subsequent infection with the virus [32]. Whether expression of integrated GP-like sequences can stimulate such cellular immunity or other types of resistance to infection remains to be explored.

## Discussion

This survey has uncovered a fossil record for currently circulating RNA virus families that stretch back some 40 million years in the evolution of host species. The error rate per replication of the DNA genomes of the hosts is much lower than the error rates of RNA-dependent RNA synthesis, the mechanism by which these viruses replicate their genomes. Consequently, the host genome contains a more accurate record of the archival genes of viruses with RNA genomes than the related present-day viruses. Considering the relatively high rate of mutation in RNA viruses, and the stringent criteria we utilized to detect homologies, what is reported here should be taken as an underestimate of such viral gene integration events. The most common events we detected derive from certain viruses that contain negative single strand RNA genomes. This might be a reflection of some unusual properties of such viruses and their hosts. For example, the viruses could have high sequence conservation or the hosts could have been selected to retain specific viral sequences that confer resistance to subsequent infection. However, the results of this search are as interesting for what was *not* found as what was found.

The endogenous viral sequences that were identified with highest confidence are all related to currently circulating viruses in the Order *Mononegavirales*, which contain single negative strand RNA genomes. Furthermore only two of the four recognized families in this Order are represented, the Bornaviruses (BDV) and Filoviruses (EBOV and MARV). In one species, zebrafish, we also found endogenous sequences related to members of a possible new Taxon in this viral Order, comprising Midway and Nyamanini viruses [33]. These results seem especially noteworthy, as the genomic insertions reported in plants and insects are all derived from viruses with plus strand RNA genomes, such as the Flaviviruses and the Picornaviruses [1], [2], [3]. Furthermore, the data presented here (Tables 3 and S1) indicate that the endogenous sequences in vertebrate genomes were likely integrated via target-primed reverse transcription of ancestral viral mRNAs by LINE elements. As all viruses produce mRNAs during active infection, the selection or retention of endogenous sequences from mainly one viral Order, is all the more striking.

The cellular location of viral replication does not appear to be a critical factor in the insertion of endogenous sequences, because the Bornaviruses replicate in the nucleus and the Filoviruses, in the cytoplasm. We note, in addition, that no endogenous sequences were found that are related to viruses in the Orthomyxovirus family, such as the influenza viruses, which contain segmented negative strand RNA genomes and also replicate in the nuclei of infected cells. However, it is possible that some feature of the mRNAs produced by these viruses is recognized preferentially by LINE machinery, or can promote access to such machinery in the nucleus, and such notions can now be tested. LINE elements are known to be active in the germline [34], and it is possible that the germline cells of some infected vertebrates may have been especially susceptible to infection by the ancestors of these viruses. Finally, DNA copies of mRNAs from other RNA viruses may, indeed, have been integrated into the germlines of infected vertebrates, but are no longer recognizable. Once DNA copies are inserted into the host genome one would expect the mutation rate of these sequences to be reduced by about four orders of magnitude compared to the genes in replicating RNA viruses, rapidly separating the virus sequences of today from the those of the past. Indeed, a DNA copy of an RNA viral genome trapped in a host chromosome is a window on the RNA virus sequences of the past. In this context, the high conservation of the BDV genome [35], [36] may partially explain our ability to detect the related endogenous sequences.

By far the most readily observable endogenous virus-like elements uncovered in our study were related to BDV. For example, these germline integrations persisted for millions of years as recognizable copies of the N gene in primate and rodent lineages, and of the N and the L genes in bats. Furthermore, an initial event appears to slow or stop further integration events, suggesting that the viral gene product(s) can inhibit further virus infection, or eliminates the need to further select for the new integration event. Several integrations also appear to have been selected for their protein coding capacity, with no stop codons emerging over the past forty million years. This is particularly striking because the amino acids in these genes appear to be undergoing the expected frequency of neutral drift, at least among shared integrations in the primate lineage.

There are several possible mechanisms by which an endogenous viral gene product may inhibit the subsequent infection of a cell or animal by the same virus. For example, synthesis from the endogenous sequence of an RNA molecule that is partially complementary to the infecting viral RNA could trigger an early interferon or RNA interference response. In addition, translation of an mRNA from the endogenous viral sequence would lead to production of a protein or peptide that is similar, but not identical to that of the infecting viral protein. In the case of nucleocapsid-like proteins (N, NP), such an endogenous gene product could block virus replication or result in the assembly of faulty, non-infectious particles. This would require genetic drift to produce missense mutations but no stop codons, which is the case for some endogenous sequences that we have discovered. Because the function of these proteins requires appropriate multimerization, even a small number of abnormal or defective, endogenously produced monomers could exert a substantial biological effect. Sequence differences in proteins expressed by the endogenous L- and VP-35-like genes could also result in assembly of defective virus particles. Such particles might then become good immunogens, providing immune protection in the host. It is also possible that production of glycoprotein peptides encoded in endogenous viral sequences might block infections by viruses with similar glycoproteins. Examples of the various resistance mechanisms cited above have been shown to exist with several virus groups. This includes experiments in rats, where ectopic expression of individual proteins of the Bornavirus N, X, and P genes, but not their mRNA, inhibits virus replication [37].

There is likely strong selection pressure to establish a resistance mechanism against Bornavirus and Ebolavirus/Marburgvirus, given their high mortality rates in susceptible species. We have noted that the natural hosts of BDV, such as cows and horses, have no detectible sequences related to the BDV N gene (Table 1), or that the integration is under no present-day selection (Table 4). It has also been reported that resistance to the neurological symptoms of BDV is genetically inherited in rats and is encoded in an unknown host gene [38]. It would now be quite interesting to test whether or not that gene is the BDV-related rodEBLN sequence. It would also be interesting to examine the endogenous sequences in the human population in greater detail, to determine if there are polymorphisms or deletions that might correlate with neurological diseases, which could lead to a re-examination of the role of BDV in such conditions.

Natural resistance to currently circulating EBOV and MARV may allow species to serve as asymptomatic reservoirs for these viruses. In microbats, we identified endogenous sequences related to the NP and VP35 genes of these Filoviruses, in addition to the N and L genes of BDV. Bats of different species have been identified as possible natural reservoirs of EBOV and MARV in areas of human outbreaks in Africa [39], [40], [41]. Recent studies confirm that these viruses co-circulate in Gabon, where bats infected by each virus are found. It should now be possible to ask if there is any correlation between the presence and properties of the endogenous sequences in the various bat species and their ability to serve as natural reservoirs for these negative strand RNA viruses.

In summary, our studies have made it clear that ancient relatives of some RNA viruses have left DNA copies of their sequences in the germline cells of their vertebrate hosts. The sources of vertebrate genetic inheritance are, therefore, considerably more diverse than previously appreciated. A number of recent reports from tissue culture experiments or clinical studies have presented evidence for the incorporation of DNA sequences corresponding to all or part of the genomes of a variety of infecting RNA viruses into host cell DNA [e.g. 5] [42], [43], indicating that such events might occur in somatic tissues with some frequency. However, the mechanisms of integration seem to be varied, and the biological impacts have yet to be elucidated. Whether the germline integrations that we have identified are simply accidents or, as we suspect, may sometimes provide the host with an important selectable advantage, can now be tested.

## Methods

Analysis of genome integrations was conducted based on viral protein sequences available at NCBI FTP website (ftp://ftp.ncbi.nih.gov/refseq/release/viral/). Most recent sequences were downloaded on October 28, 2009. A total of 79,001 sequences were included in that distribution, with each representing an individual viral protein. This number slightly overestimates the actual number of unique sequences, as some proteins may be part of a polyprotein. However, the discrepancy is small, as a total of only 561 sequences are labeled as polyproteins. Finally, every individual virus encodes more than one protein.

The complete list of viral proteins was further narrowed down to include only single stranded RNA viruses with no known DNA phase in their replication. For this purpose, we used the list in the NCBI taxonomy database, downloaded on the same date as the viral protein sequences (http://www.ncbi.nlm.nih.gov/Taxonomy/). This screening procedure yielded 5,666 independent viral protein sequences. Again, small overlap is possible due to dual representation in polyproteins.

Viral sequences were then screened against publicly available genomic assemblies of 48 sequenced vertebrates and a few close siblings. Vertebrate sequences were downloaded from the UCSC genome website, and when not available, directly from the sequencing center websites or from the Ensembl database (release 56). The list of species considered is given in Table S9. The initial search was performed using BLAST 2.2.17 with parameters -p tblastn -M BLOSUM62 -e 1e-4.

A direct search produced 14,281 results, with BLAST E-value cutoff at 10^−04^. The vast majority of hits arose from homology between viral proteins and a few host proteins. By far the most widespread homology was between the gene for a 60–70 kDa protein in plant viruses and vertebrate heat shock proteins (HSP70 in humans). Similarly, several viral genes had homologies with GIMAP8, BIRC8, PARP14, and the DNAJC14 families of genes. We removed from further consideration any viral protein that had homology with known mRNAs in humans, cows and mice at the same time. Any integrations in this group would likely represent host pseudogenes, rather than integrations of viral origin.

As a final crosscheck, all integrated sequences were reverse-searched against all known nucleotide and protein sequences in the NCBI database using BLAST algorithm, to ensure that a putative integration is indeed from a virus with a single strand RNA genome, and is not a homologous protein from another virus or organism. Additionally, all reported sequences have 30–50% identity with the present-day virus proteins. These values are common for many homologous proteins in Ensembl database, and support an evolutionary relationship between the integrated sequences we have identified and present day virus proteins.

Altogether we identified strong hits from seven viral proteins from three different viruses/families (Table 1), all within the Order *Mononegavirales* (non-segmented single stranded negative sense RNA viruses). The sole exception that resembles a Flavivirus-derived gene is discussed below. All of the *Mononegavirales*-derived hits come from nucleocapsid (N, NP), and matrix (M) proteins, as well as the viral RNA-dependent DNA polymerase (L), and the polymerase complex cofactor (VP35). Additionally, weaker hits were associated with glycoproteins (G, GP) of the same viruses. Extra care has to be taken here, as glycoproteins are encoded in many viral genomes including retroviruses, which are commonly integrated in the germ lines. We did the following checks to eliminate potential retroviral glycoproteins from further consideration: regions of 10 kb extending both downstream and upstream of each potential glycoprotein-like integration were downloaded and checked for retroviral *gag-* and *pol*-genes, as well as for LTR-signatures. Retroviral *pol* genes were chosen for their highest conservation among all retroviral genes. Altogether, *gag-* and *pol-* genes were downloaded from approximately 50 different retrovirus families, and searched using blastx algorithm of the BLAST program, with E-value threshold of 10^−3^. Search for LTR-sequences was conducted using LTR-FIND tool (http://tlife.fudan.edu.cn/ltr_finder/) [44].

While all aforementioned integrations were related to members of the *Mononegavirales*, one putative integration on scaffold 1104 of medaka is most similar to a virus with a positive strand RNA genome, the Flavivirus, Tamana Bat virus. Integration with putative coordinates 26500-2900 on scaffold 1104 has low sequence similarity to Tamana Bat virus and several other Flaviviruses. However, sequence similarity of this integration is fairly low (BLAST value 10∧-7 for a 190 amino acid fragment of a 600 amino acid protein, with sequence identity of just 28%). Additionally, the entire scaffold is not yet mapped to a chromosome, has no known genes, and is not readily aligned with other species. It therefore remains to be seen if this is an actual integration of a positive-sense virus, some accidental sequence, or the result of laboratory contamination. The possibility of somatic cell integration, as opposed to germ-line integration, also remains open, as medaka sequencing relies on genomic DNA from adult bodies [45].

## Supporting Information

Table S1 List of Endogenous Borna-Like N (EBLN) integrations
(0.13 MB DOC)

Table S2 List of Endogenous Borna-like M (EBLM) integrations
(0.03 MB DOC)

Table S3 List of Endogenous Borna-like L (EBLL) integrations
(0.10 MB DOC)

Table S4 List of Endogenous Ebola-like Nucleoprotein (EELN) integrations
(0.09 MB DOC)

Table S5 List of Endogenous Ebola-like VP35 (EEL35) integrations
(0.03 MB DOC)

Table S6 List of Endogenous Ebola-like L (EELL) integrations
(0.04 MB DOC)

Table S7 List of Endogenous Midway/Nyamanini and Tamana bat virus like integrations
(0.04 MB DOC)

Table S8 List of vertebrate integrations found by BLAST search and number of stop codons inside aligned aminoacids
(0.18 MB DOC)

Table S9 List of species and assemblies analyzed
(0.07 MB DOC)

Figure S1 Phylogeny of Filovirus-like NP gene integrations
(0.51 MB TIF)

Figure S2 Expression data for the probe 2199906 at that maps onto hsEBLN-2 integration of Borna-like p40 gene in humans [46], [47]
(0.06 MB TIF)

Figure S3 Alignments of Bornavirus matrix proteins and related endogenous sequences. The indicated endogenous sequences are compared with sequences of Bornavirus isolated from a variety of species including: horse (AJ311524), cow (AB246670), sheep (AY066023), human (AB032031). We used the default color scheme for Clustal W alignment in the Jalview program.
(1.08 MB TIF)

Figure S4 Comparison of the Bornavirus L protein sequence with Bornavirus L-like endogenous sequences. The indicated endogenous sequences are compared with sequences of Bornavirus isolated from a variety of species including: cow (AB246670), human (AB032031), horse (AJ311524), and birds (EU781967). We used the default color scheme for Clustal W alignment in the Jalview program.
(9.65 MB TIF)
