# Supplementary material for: Unexpected Inheritance: Multiple Integrations of Ancient Bornavirus and Ebolavirus/Marburgvirus Sequences in Vertebrate Genomes
Source: PLoS Pathog. 2010 Jul 29;6(7):e1001030. doi: 10.1371/journal.ppat.1001030 (PMC2912400; doi:10.1371/journal.ppat.1001030)
Supplement: Table S1 — List of Endogenous Borna-Like N (EBLN) integrations (0.13 MB DOC) [file ppat.1001030.s001.doc]

**Table S1.** ***List of Endogenous Borna-Like N (EBLN) integrations.***

| Specie | Scaffold or Chromosome | Location on scaffold | Location within Bornavirus N protein^1)^ | BLAST E‑value and percent identity | Label | Significant large ORFs (length and position) |
| --- | --- | --- | --- | --- | --- | --- |
| Human  (*Homo Sapiens*) ^2)^ | Chr10 | 22497869-22498822 | 28-349 | 2E-65 / 41% | hsEBLN-1 | 366aa (full protein) |
|  | Chr3 | 73111434-73112324 | 24-319 | 7E-40 / 33% | hsEBLN-2 | 225aa (residues TSS-225) |
|  |  | 73112206-73112361 | 280-331 | 3E-07 / 48% |  |  |
|  | Chr9 | 37086816-37087331 | 37-217 | 2E-15 / 34% | hsEBLN-3 | not found |
|  |  | 37087138-37087686 | 150-331 | 3E-30 / 40% |  |  |
|  | Chr17 | 4474548-4475132 | 70-267 | 4E-31 / 35% | hsEBLN-4 | 109aa (residues 170-279) ^3)^ |
|  |  | 4475207-4475323 | 293-331 | 4E-31 / 46% |  |  |
|  | Chr1 | 235281099-235281323 | 196-274 | 1E-08 / 30% | hsEBLN-5 | not found |
|  |  | 235280959-235281111 | 271-321 | 1E-08 / 29% |  |  |
|  | Chr11 | 74723123-74723431 | 222-323 | 1E-05 / 30% | hsEBLN-6 | 184aa (residues 217-end) ^3)^ |
|  | Chr10 | 96294130-96294423 | 227-323 | 1E-05 / 31% | hsEBLN-7 | not found |
| Squirrel (*Spermophilus Tridecemlineatus)* | Scaffold 113120 | 80449-81435 | 40-368 | 1E-155 / 77% | stEBLN | 203aa (residues 170-370) |
| Guinea Pig  (*Cavia Procellus*) | Scaffold 52 | 10801452-10801766 | 123-228 | 1E-24 / 33% | cpEBLN | not found |
|  |  | 10801760-10801885 | 227-268 | 1E-24 / 33% |  |  |
|  |  | 10801867-10802052 | 270-336 | 1E-24 / 44% |  |  |
| Mouse  (Mus Musculus) | Chr11 | 103490264-103490965 | 50-286 | 1E-47 / 37% | rodEBLN-1 | 139aa (residues 170-308) ^3)^ |
|  |  | 103490037-103490273 | 298-370 | 1E-47 / 37% |  |  |
|  | Chr11 | 93851147-93851731 | 100-300 | 2E-17 / 34% | rodEBLN-2 | not found |
|  |  | 93851632-93851916 | 271-367 | 3E-11 / 40% |  |  |
|  | Chr18 | 67195203-67195649 | 50-195 | 4E-42 / 31% | rodEBLN-3 | 98aa (residues 249-346) ^3)^ |
|  |  | 67195652-67195732 | 197-223 | 4E-42 / 51% |  |  |
|  |  | 67195762-67196046 | 233-327 | 4E-42 / 42% |  |  |
|  |  | 67196040-67196180 | 324-370 | 4E-42 / 40% |  |  |
|  | Chr6 | 87894228-87894689 | 50-204 | 1E-45 / 38% | rodEBLN-4 | 122aa (residues 86-208) |
|  |  | 87894691-87895035 | 205-327 | 1E-45 / 43% |  |  |
|  | Chr9 | 54093749-54093919 | 218-274 | 5E-08 / 56% | rodEBLN-5 | not found |
| Rat  (*Rattus Norvegicus*) | Chr10 | 92785362-92785688 | 43-151 | 5E-062 / 41% | rodEBLN-1 | not found |
|  |  | 92784712-92785251 | 190-369 | 5E-062 / 45% |  |  |
|  | Chr10 | 82624298-82624948 | 28-243 | 9E-031 / 33% | rodEBLN-2 | not found |
|  |  | 82624951-82625334 | 245-370 | 9E-031 / 32% |  |  |
|  | Chr18 | 63553420-63553629 | 28-97 | 4E-06 / 41% | rodEBLN-3 | 197aa (residues 121-319) ^3)^ |
|  |  | 63553599-63554192 | 93-296 | 4E-025 / 37% |  |  |
|  | Chr4 | 122161250-122161456 | 207-276 | 2E-026 / 50% | rodEBLN-4 | not found |
|  |  | 122161446-122161697 | 273-356 | 2E-026 / 41% |  |  |
|  | Chr8 | 57747079-57747381 | 176-274 | 4E-018 / 46% | rodEBLN-5 | not found |
| Opossum (*Monodelphis Domestica*) | Chr1 | 685483194-685483517 | 45-145 | 7E-24 / 33% | mdEBLN-1 | not found |
|  |  | 685483492-685484079 | 144-346 | 7E-24 / 31% |  |  |
|  | Chr6 | 63413280-63413567 | 163-261 | 1E-12 / 37% | mdEBLN-2 | not found |
| Cow (*Bos Taurus)* | Chr8 | 17765732-17766307 | 127-323 | 3E-07 / 28% | btEBLN | not found |
| Shrew ^4)^  (*Sorex Araneus*) | Scaffold 210981 | 4690-5190 | 161-323 | 4E-11 / 31% | saEBLN |  |
|  | Scaffold 254229 | 430-885 | 159-305 | 3E-09 / 31% |  |  |
| Wallaby ^4)^ (*Macropus Eugenii*) | Scaffold 84491 | 1029-1433 | 202-336 | 2E-07 / 33% | meEBLN | not found |
| Tarsier ^4)^  (*Tarsius Syrichta*) | Scaffold 45563 | 1561-1671 | 166-213 | 5E-09 / 31% | tsEBLN |  |
|  |  | 1284-1562 | 216-311 | 5E-09 / 39% |  |  |
| Lemur ^4)^  (*Microcebus Murinus*) | Scaffold 5488 | 24453-24728 | 4-98 | 3E-027 / 32% | mimEBLN |  |
|  |  | 23968-24453 | 103-262 | 3E-027 / 36% |  |  |
|  |  | 23846-23914 | 280-302 | 3E-027 / 43% |  |  |
| Microbat ^4)^  (*Myotis Lucifugus*) | GeneScaffold 3587 | 92858-93361 | 161-323 | 3E-12 / 29% | mlEBLN-1 | 367aa (full protein) ^3)^ |
|  | GeneScaffold 3610 | 129501-129869 | 188-305 | 2E-09 / 32% | mlEBLN-2 | 122aa (residues 209-329) ^3)^ |
|  | Scaffold 139816 | 81605-82000 | 209-336 | 2E-13 / 37% | mlEBLN-3 |  |
|  | Scaffold 174441 | 20397-20687 | 220-316 | 4E-08 / 38% | mlEBLN-4 | 108aa (residues 222-319) |
|  | Scaffold 146322 | 514-780 | 219-306 | 5E-09 / 37% | mlEBLN-5/A |  |
|  | Scaffold 20240 | 919-1185 | 219-306 | 5E-09 / 37% | mlEBLN-5/B |  |
| Bushbaby ^4)^ (*Otolemur Garnettii*) | Scaffold 101428 | 54844-55347 | 161-323 | 1E-12 / 29% | ogEBLN-1 |  |
|  | Scaffold 110219 | 228036-228371 | 70-187 | 1E-27 / 32% | ogEBLN-2 |  |
|  |  | 227813-228061 | 180-262 | 1E-27 / 36% |  |  |
|  |  | 227619-227750 | 284-326 | 1E-27 / 40% |  |  |
|  | Scaffold 16089 | 7738-7989 | 192-275 | 6E-07 / 34% | ogEBLN-3 |  |
|  |  | 8024-8185 | 288-342 | 6E-07 / 34% |  |  |
| Elephant ^4)^ (*Loxodonta Africana*) | Scaffold 15079 | 11672-11869 | 45-109 | 4E-29 / 43% | laEBLN-1 |  |
|  |  | 11929-12297 | 128-251 | 4E-29 / 43% |  |  |
|  | Scaffold 5397 | 66566-67333 | 103-358 | 2E-24 / 32% | laEBLN-2 |  |
|  | Scaffold 2021 | 69511-69663 | 69-119 | 2E-09 / 48% | laEBLN-3 |  |
|  |  | 69698-70087 | 128-257 | 2E-09 / 31% |  |  |
|  |  | 69894-70424 | 194-369 | 8E-12 / 30% |  |  |
|  | Scaffold 9702 | 42716-42889 | 51-107 | 3E-11 / 39% | laEBLN-4 |  |
|  |  | 42870-43211 | 102-221 | 3E-11 / 27% |  |  |
|  | Scaffold 1343 | 73221-73607 | 202-329 | 2E-08 / 30% | laEBLN-5 |  |
|  | Scaffold 7441 | 11567-11977 | 200-336 | 2E-07 / 30% | laEBLN-6 |  |
| Kangaroo Rat ^4)^ (*Dipodimus Ordii*) | GeneScaffold 1030 | 10554-10862 | 219-320 | 4E-07 / 28% | doEBLN |  |
| Lamprey ^4)^ (*Petromyzon Marinus*) | Contig 20784 | 8668-9033 | 197-318 | 2E-17 / 40% | pmEBLN |  |

^1)^ Full protein length is 370 aminoacids

^2)^ Only human insertions in the primate lineage are shown. List of homologous integrations in other primates can be found in Horie et al (5)

^3)^ Open reading frames may extend beyond amino acid alignments by BLAST program. In this column we report extrapolated boundaries of open reading frames.

^4)^ Assemblies mapped to scaffolds are generally preliminary, and are prone to high rates of error in base calls. Number of actual integrations may be significantly smaller than number of scaffolds reported in the table. We generally do not report open reading frames for these assemblies.
