# Supplementary material for: Unexpected Inheritance: Multiple Integrations of Ancient Bornavirus and Ebolavirus/Marburgvirus Sequences in Vertebrate Genomes
Source: PLoS Pathog. 2010 Jul 29;6(7):e1001030. doi: 10.1371/journal.ppat.1001030 (PMC2912400; doi:10.1371/journal.ppat.1001030)
Supplement: Table S2 — List of Endogenous Borna-like M (EBLM) integrations (0.03 MB DOC) [file ppat.1001030.s002.doc]

**Table S2.** List of Endogenous Borna-like M (EBLM) integrations*.

| Specie | Scaffold | Location on scaffold | Location within Bornavirus M protein^1)^ | BLAST E‑value and percent identity | Label | Significant large ORFs (length and position) |
| --- | --- | --- | --- | --- | --- | --- |
| Grey Mouse Lemur (*Microcebus Murinus*) | Scaffold 5488 | 23287-23541 | 1-94 | 4E-14 / 43% | mmEBLM | 93aa (residues TSS-102) ^2)^ |
|  |  | 23201-23281 | 97-123 | 4E-14 / 51% |  |  |
| Medaka *(Oryzias Latipes)* | Scaffold 1213 | 3892-4233 | 15-138 | 5E-07 / 33% | olEBLM | 69aa (residues TSS-71) |

^1)^ Full protein length is 142 aminoacids.

^2)^ Open reading frames may extend beyond amino acid alignments by BLAST program. In this column we report extrapolated boundaries of open reading frames.
