# Supplementary material for: Unexpected Inheritance: Multiple Integrations of Ancient Bornavirus and Ebolavirus/Marburgvirus Sequences in Vertebrate Genomes
Source: PLoS Pathog. 2010 Jul 29;6(7):e1001030. doi: 10.1371/journal.ppat.1001030 (PMC2912400; doi:10.1371/journal.ppat.1001030)
Supplement: Table S3 — List of Endogenous Borna-like L (EBLL) integrations (0.10 MB DOC) [file ppat.1001030.s003.doc]

**Table S3.** ***List of Endogenous Borna-like L (EBLL) integrations.***

| Specie | Scaffold or Chromosome | Location on scaffold or chromosome | Location within Bornavirus L protein ^1)^ | BLAST E‑value and percent identity | Label | Significant large ORFs (length and position) |
| --- | --- | --- | --- | --- | --- | --- |
| Medaka *(Oryzias Latipes)* | Scaffold 1104 | 16811-17056 | 432-512 | 8E-34 / 53% | olEBLL | 86aa (residues 519-605) ^3)^  68aa (residues 592-658) ^3)^ |
|  |  | 16527-16802 | 516-608 | 8E-34 / 44% |  |  |
|  |  | 15611-15730 | 619-657 | 1E-14 / 47% |  |  |
|  |  | 15354-15611 | 660-751 | 1E-14 / 32% |  |  |
|  |  | 15227-15343 | 755-793 | 1E-14 / 46% |  |  |
| Takifugu (*Takifugu Rubripes)* | chrUn | 259570580-259570858 | 339-431 | 4E-77 / 35% | trEBLL |  |
|  |  | 259570465-259570581 | 431-469 | 4E-77 / 51% |  |  |
|  |  | 259570197-259570478 | 465-561 | 4E-77 / 49% |  |  |
|  |  | 259569734-259570162 | 576-718 | 4E-77 / 46% |  |  |
|  |  | 259569454-259569804 | 700-814 | 3E-09 / 38% |  |  |
| Mouse (Mus Musculus) | chr19 | 30608762-30609868 | 291-657 | 3E-60 / 27% | rodEBLL | 96aa (residues 434-527)  82aa (residues 798-879) ^3)^  99aa (residues 902-1001) |
|  |  | 30609889-30610515 | 665-875 | 3E-60 / 30% |  |  |
|  |  | 30610545-30610889 | 886-1001 | 3E-60 / 29% |  |  |
| Rat (*Rattus Norvegicus*) | chr1 | 234382705-234383934 | 396-811 | 6E-78 / 37% | rodEBLL | 108aa (residues 820-926) ^3)^ |
|  |  | 234383964-234384260 | 828-925 | 6E-78 / 37% |  |  |
|  |  | 234384298-234384690 | 940-1074 | 6E-78 / 28% |  |  |
| Microbat ^3)^ (*Myotis Lucifugus*) | Scaffold 114379 | 6276-6644 | 186-307 | 3E-96 / 36% | mlEBLL-1/A | poor genome assembly,  >170aa and 149aa ORFs likely |
|  |  | 5909-6235 | 323-431 | 3E-96 / 33% |  |  |
|  |  | 5706-5909 | 431-498 | 3E-96 / 48% |  |  |
|  |  | 4996-5646 | 518-735 | 3E-96 / 47% |  |  |
|  | Scaffold 114379 | 2999-3685 | 189-431 | 4E-92 / 30% | mlEBLL-1/B |  |
|  |  | 2751-2999 | 431-512 | 4E-92 / 46% |  |  |
|  |  | 2104-2745 | 515-729 | 4E-92 / 48% |  |  |
|  |  | 431-1033 | 731-919 | 2E-40 / 33% |  |  |
|  |  | 1-393 | 935-1066 | 2E-40 / 35% |  |  |
|  | GeneScaffold 6068 | 10331-10786 | 189-342 | 1E-21 / 37% | mlEBLL-1/C |  |
|  |  | 9591-10169 | 542-735 | 1E-42 / 46% |  |  |
|  |  | 8268-8534 | 731-819 | 4E-53 / 33% |  |  |
|  |  | 7900-8172 | 841-930 | 4E-53 / 38% |  |  |
|  |  | 7344-7895 | 935-1119 | 4E-53 / 34% |  |  |
|  |  | 7082-7258 | 1152-1210 | 4E-53 / 30% |  |  |
|  | Scaffold 127324 | 3676-4785 | 593-966 | 1E-75 / 45% | mlEBLL-1/D |  |
|  | Scaffold 114706 | 183-581 | 384-512 | 1E-66 / 38% | mlEBLL-1/E |  |
|  |  | 587-760 | 515-569 | 1E-66 / 48% |  |  |
|  |  | 802-1245 | 588-735 | 1E-66 / 50% |  |  |
|  | Scaffold 140061 | 38872-39132 | 957-1044 | 8E-26 / 38% | mlEBLL-1/F |  |
|  |  | 38388-38861 | 1049-1209 | 8E-26 / 31% |  |  |
|  | GeneScaffold 3610 | 130962-131168 | 810-878 | 5E-39 / 49% | mlEBLL-2/A |  |
|  |  | 131167-131907 | 879-1129 | 5E-39 / 32% |  |  |
|  | GeneScaffold 6068 | 963-1649 | 853-1083 | 2E-38 / 37% | mlEBLL-2/B |  |
|  | Scaffold 166686 | 4653-5087 | 186-330 | 7E-08 / 29% | mlEBLL-3 |  |
|  | Scaffold 112244 | 3794-4012 | 289-362 | 1E-28 / 44% | mlEBLL-4 |  |
|  |  | 3412-3732 | 384-492 | 1E-28 / 42% |  |  |
|  | Scaffold 172127 | 38345-38719 | 958-1083 | 1E-15 / 35% | mlEBLL-5 |  |
| Wallaby ^3)^ (*Macropus Eugenii*) | Scaffold 1715 | 48932-49138 | 478-553 | 2E-43 / 28% | meEBLL-1 | 120aa (residues 873-992) ^2)^ |
|  |  | 48802-48936 | 552-601 | 2E-43 / 32% |  |  |
|  |  | 48615-48773 | 628-681 | 2E-43 / 48% |  |  |
|  |  | 48194-48553 | 714-832 | 2E-43 / 44% |  |  |
|  |  | 47959-48216 | 826-919 | 2E-43 / 37% |  |  |
|  | Scaffold13330 | 7189-7224 | 549-560 | 6E-13 / 75% | meEBLL-2 | not found |
|  |  | 6692-7168 | 570-732 | 6E-13 / 30% |  |  |
|  | Scaffold100180 | 3696-4208 | 396-569 | 1E-12 / 35% | meEBLL-3 | not found |

^1)^ Full protein length is 1608 aminoacids.

^2)^ Open reading frames may extend beyond amino acid alignments by BLAST program. In this column we report extrapolated boundaries of open reading frames.

^3)^ Assemblies mapped to scaffolds are generally preliminary, and are prone to high rates of error in base calls. Number of actual integrations may be significantly smaller than number of scaffolds reported in the table. We generally do not report open reading frames for these assemblies.
