# Supplementary material for: Unexpected Inheritance: Multiple Integrations of Ancient Bornavirus and Ebolavirus/Marburgvirus Sequences in Vertebrate Genomes
Source: PLoS Pathog. 2010 Jul 29;6(7):e1001030. doi: 10.1371/journal.ppat.1001030 (PMC2912400; doi:10.1371/journal.ppat.1001030)
Supplement: Table S4 — List of Endogenous Ebola-like Nucleoprotein (EELN) integrations (0.09 MB DOC) [file ppat.1001030.s004.doc]

**Table S4.** ***List of Endogenous Ebola-like Nucleoprotein (EELN) integrations.***

| Specie | Scaffold or Chromosome | Most similar virus strain^1)^ | Location on scaffold or chromosome | Location within EBOV/MARV N protein ^2)^ | BLAST E value and percent identity | Label | Significant large ORFs (length and position) |
| --- | --- | --- | --- | --- | --- | --- | --- |
| Microbat^3)^ (*Myotis Lucifugus*) | Scaffold131047 | Lake Victoria Marburgvirus | 79-537 | 63-214 | 2E-36 / 27% | mlEELN-1 | 158aa (residues 72-228) ^4)^ and 164aa (residues 228-391) |
|  |  |  | 537-1187 | 215-437 | 2E-36 / 35% |  |  |
|  | Scaffold140100 | Zaire Ebolavirus | 102678-103811 | 30-403 | 2E-17 / 26% | mlEELN-2 |  |
|  | Scaffold174657 | Sudan Ebolavirus | 33786-34073 | 160-255 | 3E-28 / 36% | mlEELN-3 | not found |
|  |  |  | 33463-33765 | 263-363 | 3E-28 / 47% |  |  |
|  | GeneScaffold222 | Lake Victoria Marburgvirus | 5576-5854 | 223-315 | 3E-18 / 40% | mlEELN-4 | 117aa (residues 201-317) ^4)^ |
|  |  |  | 5290-5583 | 314-418 | 3E-18 / 26% |  |  |
| Shrew (Sorex Araneus) | Scaffold140193 | Lake Victoria Marburgvirus | 4760-5113 | 170-308 | 4E-08 / 29% | saEELN | 136aas (residues 166-322) ^4)^ |
| Guinea Pig (*Cavia Porcellus*) | Scaffold 20 | Lake Victoria Marburgvirus | 30924930-30925232 | 224-321 | 1E-08 / 35% | cpEELN | not found |
| Opossum (Monodelphis Domestica) | chr2 | Reston Ebolavirus | 54443969-54444142 | 175-231 | 4E-39 / 37% | mdEELN | not found |
|  |  |  | 54444145-54444399 | 232-316 | 4E-39 / 55% |  |  |
|  |  |  | 54444387-54444668 | 316-409 | 4E-39 / 44% |  |  |
| Wallaby ^3)^ (*Macropus Eugenii*) | Scaffold10584 | Lake Victoria Marburgvirus | 11568-11705 | 98-146 | 9E-53 / 48% | meEELN-1 | not found |
|  |  |  | 11296-11556 | 151-237 | 9E-53 / 54% |  |  |
|  |  |  | 10929-11279 | 243-361 | 9E-53 / 48% |  |  |
|  | Scaffold108552 | Lake Victoria Marburgvirus | 6694-6882 | 274-336 | 7E-13 / 65% | meEELN-2 | not found |
|  | Scaffold10890 | Reston Ebolavirus | 15530-15655 | 149-190 | 3E-26 / 57% | meEELN-3 | not found |
|  |  |  | 15666-15896 | 197-274 | 3E-26 / 50% |  |  |
|  |  |  | 15907-16008 | 281-314 | 3E-26 / 52% |  |  |
|  | Scaffold115193 | Sudan Ebolavirus | 970-1512 | 68-248 | 1E-39 / 39% | meEELN-4 | not found |
|  |  |  | 693-965 | 251-343 | 1E-39 / 46% |  |  |
|  | Scaffold117569 | Sudan Ebolavirus | 68-907 | 22-312 | 1E-28 / 33% | meEELN-5 | >218aa likely (incomplete scaffold) |
|  | Scaffold155090 | Zaire Ebolavirus | 6556-7056 | 56-237 | 5E-14 / 27% | meEELN-6 | not found |
|  |  |  | 6416-6556 | 240-286 | 5E-14 / 44% |  |  |
|  |  |  | 6351-6419 | 287-309 | 5E-14 / 56% |  |  |
|  | Scaffold16638 | Reston Ebolavirus | 2637-2783 | 64-112 | 2E-05 / 32% | meEELN-7 | not found |
|  |  |  | 2450-2533 | 148-175 | 2E-05 / 64% |  |  |
|  |  |  | 1997-2374 | 258-389 | 2E-15 / 40% |  |  |
|  | Scaffold197700 | Sudan Ebolavirus | 1262-1789 | 60-233 | 2E-18 / 27% | meEELN-8 | not found |
|  |  |  | 816-1241 | 241-387 | 2E-18 / 26% |  |  |
|  | Scaffold320693 | Lake Victoria Marburgvirus | 157-342 | 71-132 | 1E-39 / 27% | meEELN-9 | not found |
|  |  |  | 360-575 | 139-211 | 1E-39 / 42% |  |  |
|  |  |  | 574-1038 | 213-367 | 1E-39 / 45% |  |  |
|  | Scaffold44816 | Sudan Ebolavirus | 1867-2058 | 130-193 | 1E-05 / 40% | meEELN-10 | not found |
|  |  |  | 2121-2528 | 250-384 | 1E-17 / 43% |  |  |
|  | Scaffold70101 | Zaire Ebolavirus | 7234-7521 | 56-151 | 9E-30 / 33% | meEELN-11 | 142aa(?); coding sequence disrupted by more recent integration |
|  |  |  | 6932-7204 | 164-254 | 9E-30 / 52% |  |  |
|  |  |  | 4085-4138 | 249-266 | 5E-10 / 55% |  |  |
|  |  |  | 3789-4076 | 273-368 | 5E-10 / 39% |  |  |
|  | Scaffold99538 | Sudan Ebolavirus | 5690-6457 | 136-392 | 4E-43 / 39% | meEELN-12 | not found |

^1)^ In general, it is not possible to uniquely associate integration with individual Ebola strain. Most similar strain is displayed to identify the refseq sequence used to map coordinates and to calculate BLAST E-value.

^2)^ Full protein length is 738/739 aminoacids in Ebolavirus, and 695 aminoacids in Marburgvirus.

^3)^ Assemblies mapped to scaffolds are generally preliminary, and are prone to high rates of error in base calls. Number of actual integrations may be significantly smaller than number of scaffolds reported in the table. We generally do not report open reading frames for these assemblies.

^4)^ Open reading frames may extend beyond amino acid alignments by BLAST program. In this column we report extrapolated boundaries of open reading frames.
