# Supplementary material for: Unexpected Inheritance: Multiple Integrations of Ancient Bornavirus and Ebolavirus/Marburgvirus Sequences in Vertebrate Genomes
Source: PLoS Pathog. 2010 Jul 29;6(7):e1001030. doi: 10.1371/journal.ppat.1001030 (PMC2912400; doi:10.1371/journal.ppat.1001030)
Supplement: Table S5 — List of Endogenous Ebola-like VP35 (EEL35) integrations (0.03 MB DOC) [file ppat.1001030.s005.doc]

***Table S5.*** List of Endogenous Ebola-like VP35 (EEL35) integrations.

| Specie | Scaffold | Most similar virus strain ^1)^ | Location on scaffold | Location within Ebolavirus VP35 protein ^1)^ | BLAST E-value and percent identity | Label | Significant large ORFs (length and position) |
| --- | --- | --- | --- | --- | --- | --- | --- |
| Microbat (*Myotis Lucifugus*) | Scaffold144630 | Reston Ebolavirus | 9870-10658 | 74-329 | 5E-23 / 30% | mlEEL35 | 281aa (residues 52-329) |
| Tarsier (*Tarsius Syrichta*) | Scaffold_521 | Reston Ebolavirus | 86926-87291 | 138-253 | 5E-16 / 29% | tsEEL35 | 131aa (residues 137-261) ^2)^ |
|  |  |  | 86724-86930 | 261-329 | 5E-16 / 42% |  |  |
| Wallaby (*Macropus Eugenii*) | Scaffold42523 | Reston Ebolavirus | 226-618 | 94-227 | 3E-15 / 24% | meEEL35 | not found (incomplete scaffold) |
|  |  |  | 12-221 | 231-300 | 3E-15 / 45% |  |  |

^1)^ Full protein length is 329 aminoacids in the Reston Strain.

^2)^ Open reading frames may extend beyond amino acid alignments by BLAST program. In this column we report extrapolated boundaries of open reading frames.
