# Supplementary material for: Unexpected Inheritance: Multiple Integrations of Ancient Bornavirus and Ebolavirus/Marburgvirus Sequences in Vertebrate Genomes
Source: PLoS Pathog. 2010 Jul 29;6(7):e1001030. doi: 10.1371/journal.ppat.1001030 (PMC2912400; doi:10.1371/journal.ppat.1001030)
Supplement: Table S6 — List of Endogenous Ebola-like L (EELL) integrations (0.04 MB DOC) [file ppat.1001030.s006.doc]

***Table S6.*** List of Endogenous Ebola-like L (EELL) integrations.

| Specie | Chromosome | Most similar virus strain^1)^ | Location on chromosome | Location within Marburgvirus L protein ^1)^ | BLAST E‑value and percent identity | Label | Significant large ORFs (length and position) |
| --- | --- | --- | --- | --- | --- | --- | --- |
| Opossum (Monodelphis Domestica) | chr3 | Lake Victoria Marburgvirus | 99735005-99735253 | 605-687 | 5E-72 / 51% | mdEELL | not found |
|  |  |  | 99734704-99735003 | 691-790 | 5E-72 / 32% |  |  |
|  |  |  | 99734268-99734675 | 806-943 | 5E-72 / 41% |  |  |
|  |  |  | 99734138-99734266 | 945-988 | 5E-72 / 43% |  |  |
|  |  |  | 99733903-99734133 | 991-1067 | 5E-72 / 44% |  |  |
|  |  |  | 99732236-99732451 | 1282-1354 | 7E-12 / 58% |  |  |

^1)^ Full protein length is 2331 aminoacids.
