# Supplementary material for: Unexpected Inheritance: Multiple Integrations of Ancient Bornavirus and Ebolavirus/Marburgvirus Sequences in Vertebrate Genomes
Source: PLoS Pathog. 2010 Jul 29;6(7):e1001030. doi: 10.1371/journal.ppat.1001030 (PMC2912400; doi:10.1371/journal.ppat.1001030)
Supplement: Table S7 — List of Endogenous Midway/Nyamanini and Tamana bat virus like integrations (0.04 MB DOC) [file ppat.1001030.s007.doc]

***Table S7.*** List of endogenous Midway/Nyamanini and Tamana bat virus like integrations.

| Specie | Scaffold or Chromosome | Integrated protein | Location on scaffold or chromosome | Location within Ebola VP35 protein | BLAST E-value and percent identity | Label | Significant large ORFs (length and position) |
| --- | --- | --- | --- | --- | --- | --- | --- |
| Medaka (*Oryzias Latipes*) | scaffold1104 | NS3-like protein, Tamana Bat Virus | 27200-27769 | 271-464 | 2E-07 / 28% | olENS3 | 212aa (residues 313-526) ^2)^ |
| Zebrafish (*Danio Rerio*) | chr7 | L-protein,  Midway Virus | 5403187-5404776 | 381-924 | 6E-025 / 23% | drEMLL-1 | 161aa (residues 405-568)  319aa (residues 659-980) ^2)^ |
|  | chr20 | L-protein,  Midway Virus | 10806996-10809302 | 362-1142 | 5E-032 / 21% | drEMLL-2 | 590aa (residues 351-953) ^2)^ |
|  | chr25 | L-protein,  Midway Virus | 23545949-23548084 | 238-962 | 8E-027 / 21% | drEMLL-3 | 761aa (residues TSS-756) ^2)^  180aa (residues 792-971) ^2)^ |
|  | chr4 | L-protein,  Midway Virus | 66339375-66340469 | 231-593 | 9E-14 / 22% | drEMLL-4 |  |
|  | chr4 | L-protein,  Midway Virus | 42016876-42017517 | 475-697 | 7E-08 / 24% | drEMLL-5 |  |
|  | Zv8_scaffold3025 | L-protein,  Midway Virus | 269149-270384 | 541-968 | 3E-13 / 20% | drEMLL-6 |  |

^1)^ Full protein length is 651 aminoacids fro L-protein of Midway virus, and 624 aminoacids for NS3 proteins of the Tamana Bat virus.

^2)^ Open reading frames may extend beyond amino acid alignments by BLAST program. In this column we report extrapolated boundaries of open reading frames.
