# Supplementary material for: Unexpected Inheritance: Multiple Integrations of Ancient Bornavirus and Ebolavirus/Marburgvirus Sequences in Vertebrate Genomes
Source: PLoS Pathog. 2010 Jul 29;6(7):e1001030. doi: 10.1371/journal.ppat.1001030 (PMC2912400; doi:10.1371/journal.ppat.1001030)
Supplement: Table S8 — List of vertebrate integrations found by BLAST search and number of stop codons inside aligned aminoacids (0.18 MB DOC) [file ppat.1001030.s008.doc]

**Table S8.** ***List of vertebrate integrations found by BLAST search and number of stop codons inside aligned aminoacids*.***

| Integration | Specie | Virus | Integrated gene | Total number of stop codons | Total length of BLAST alignments | Sequence identity | Number of stop codons per 100 aminoacids |
| --- | --- | --- | --- | --- | --- | --- | --- |
| drEMLL-4 | Zebrafish | Midway Virus | L | 0 | 365 | 22% | 0.0 |
| stEBLN | Squirrel | Bornavirus | N | 0 | 329 | 77% | 0.0 |
| hsEBLN-1 | Human | Bornavirus | N | 0 | 318 | 41% | 0.0 |
| mlEEL35 | Microbat | Ebola/Marburgvirus | VP35 | 0 | 263 | 30% | 0.0 |
| laEBLN-2 | Elephant | Bornavirus | N | 0 | 256 | 32% | 0.0 |
| mlEBLL-2B | Microbat | Bornavirus | L | 0 | 229 | 37% | 0.0 |
| tsEEL35 | Tarsier | Ebola/Marburgvirus | VP35 | 0 | 191 | 34% | 0.0 |
| olENS3 | Medaka | Tamana Bat Virus | NS3 | 0 | 190 | 28% | 0.0 |
| ogEBLN-1 | Bushbaby | Bornavirus | N | 0 | 168 | 29% | 0.0 |
| mlEBLN-1 | Microbat | Bornavirus | N | 0 | 168 | 29% | 0.0 |
| saEBLN-1 | Shrew | Bornavirus | N | 0 | 167 | 31% | 0.0 |
| ogEBLN-3 | Bushbaby | Bornavirus | N | 0 | 138 | 34% | 0.0 |
| mlEBLN-3 | Microbat | Bornavirus | N | 0 | 132 | 37% | 0.0 |
| laEBLN-5 | Elephant | Bornavirus | N | 0 | 129 | 30% | 0.0 |
| mlEBLL-5 | Microbat | Bornavirus | L | 0 | 125 | 35% | 0.0 |
| mlEBLN-2 | Microbat | Bornavirus | N | 0 | 123 | 32% | 0.0 |
| hsEBLN-6 | Human | Bornavirus | N | 0 | 103 | 30% | 0.0 |
| rodEBLN-5 | Rat | Bornavirus | N | 0 | 101 | 46% | 0.0 |
| mlEBLN-4 | Microbat | Bornavirus | N | 0 | 97 | 38% | 0.0 |
| mlEBLN-5A | Microbat | Bornavirus | N | 0 | 89 | 37% | 0.0 |
| mlEBLN-5B | Microbat | Bornavirus | N | 0 | 89 | 37% | 0.0 |
| meEELN-2 | Wallaby | Ebola/Marburgvirus | NP | 0 | 70 | 65% | 0.0 |
| drEMLL-3 | Zebrafish | Midway Virus | L | 1 | 712 | 21% | 0.1 |
| drEMLL-1 | Zebrafish | Midway Virus | L | 1 | 530 | 23% | 0.2 |
| rodEBLN-4 | Mouse | Bornavirus | N | 1 | 269 | 40% | 0.4 |
| rodEBLN-3 | Rat | Bornavirus | N | 1 | 263 | 38% | 0.4 |
| drEMLL-2 | Zebrafish | Midway Virus | L | 3 | 769 | 21% | 0.4 |
| mlEBLL-1F | Microbat | Bornavirus | L | 1 | 245 | 33% | 0.4 |
| mlEELN-3 | Microbat | Ebola/Marburgvirus | NP | 1 | 204 | 42% | 0.5 |
| mlEELN-4 | Microbat | Ebola/Marburgvirus | NP | 1 | 189 | 33% | 0.5 |
| rodEBLN-3 | Mouse | Bornavirus | N | 2 | 314 | 37% | 0.6 |
| rodEBLN-1 | Mouse | Bornavirus | N | 2 | 313 | 37% | 0.6 |
| trEBLL | Fugu | Bornavirus | L | 3 | 458 | 43% | 0.7 |
| mlEBLL-1D | Microbat | Bornavirus | L | 3 | 370 | 45% | 0.8 |
| mlEELN-1 | Microbat | Ebola/Marburgvirus | NP | 3 | 370 | 32% | 0.8 |
| hsEBLN-5 | Human | Bornavirus | N | 1 | 122 | 31% | 0.8 |
| olEBLL | Medaka | Bornavirus | L | 3 | 340 | 44% | 0.9 |
| mmEBLM | Lemur | Bornavirus | M | 1 | 112 | 45% | 0.9 |
| drEMLL-5 | Zebrafish | Midway Virus | L | 2 | 214 | 24% | 0.9 |
| cpEBLN | Guineapig | Bornavirus | N | 2 | 207 | 41% | 1.0 |
| drEMLL-6 | Zebrafish | Midway Virus | L | 4 | 412 | 20% | 1.0 |
| hsEBLN-2 | Human | Bornavirus | N | 3 | 309 | 38% | 1.0 |
| meEELN-7 | Wallaby | Ebola/Marburgvirus | NP | 2 | 203 | 41% | 1.0 |
| rodEBLN-1 | Rat | Bornavirus | N | 3 | 289 | 43% | 1.0 |
| mlEBLL-1C | Microbat | Bornavirus | L | 8 | 763 | 38% | 1.0 |
| meEELN-5 | Wallaby | Ebola/Marburgvirus | NP | 3 | 280 | 33% | 1.1 |
| meEBLL-1 | Wallaby | Bornavirus | L | 4 | 364 | 39% | 1.1 |
| mlEBLL-4 | Microbat | Bornavirus | L | 2 | 180 | 43% | 1.1 |
| rodEBLN-2 | Mouse | Bornavirus | N | 3 | 266 | 39% | 1.1 |
| meEELN-1 | Wallaby | Ebola/Marburgvirus | NP | 3 | 250 | 50% | 1.2 |
| mlEBLL-2A | Microbat | Bornavirus | L | 4 | 316 | 36% | 1.3 |
| meEELN-3 | Wallaby | Ebola/Marburgvirus | NP | 2 | 153 | 52% | 1.3 |
| saEBLN-2 | Shrew | Bornavirus | N | 2 | 152 | 31% | 1.3 |
| mlEBLL-1B | Microbat | Bornavirus | L | 13 | 857 | 38% | 1.5 |
| mdEELL | Opossum | Ebola/Marburgvirus | L | 8 | 520 | 31% | 1.5 |
| tsEBLN | Tarsier | Bornavirus | N | 2 | 130 | 37% | 1.5 |
| meEELN-11 | Wallaby | Ebola/Marburgvirus | NP | 5 | 295 | 42% | 1.7 |
| hsEBLN-4 | Human | Bornavirus | N | 4 | 234 | 37% | 1.7 |
| ogEBLN-2 | Bushbaby | Bornavirus | N | 4 | 231 | 35% | 1.7 |
| olEBLM | Medaka | Bornavirus | M | 2 | 114 | 33% | 1.8 |
| mimEBLN | Lemur | Bornavirus | N | 5 | 277 | 35% | 1.8 |
| laEBLN-4 | Elephant | Bornavirus | N | 3 | 166 | 31% | 1.8 |
| rodEBLL | Rat | Bornavirus | L | 12 | 640 | 35% | 1.9 |
| mlEBLL-1A | Microbat | Bornavirus | L | 10 | 513 | 42% | 1.9 |
| meEEL35 | Wallaby | Ebola/Marburgvirus | VP35 | 4 | 201 | 31% | 2.0 |
| hsEBLN-7 | Human | Bornavirus | N | 2 | 97 | 31% | 2.1 |
| hsEBLN-3 | Human | Bornavirus | N | 6 | 288 | 43% | 2.1 |
| mdEBLN-2 | Opossum | Bornavirus | N | 2 | 96 | 37% | 2.1 |
| meEELN-4 | Wallaby | Ebola/Marburgvirus | NP | 6 | 272 | 41% | 2.2 |
| meEBLN | Wallaby | Bornavirus | N | 3 | 135 | 33% | 2.2 |
| rodEBLL | Mouse | Bornavirus | L | 16 | 693 | 28% | 2.3 |
| mdEBLN-1 | Opossum | Bornavirus | N | 7 | 302 | 32% | 2.3 |
| laEBLN-3 | Elephant | Bornavirus | N | 7 | 297 | 33% | 2.4 |
| meEELN-10 | Wallaby | Ebola/Marburgvirus | NP | 5 | 200 | 42% | 2.5 |
| mdEELN | Opossum | Ebola/Marburgvirus | NP | 6 | 237 | 46% | 2.5 |
| btEBLN | Cow | Bornavirus | N | 5 | 192 | 28% | 2.6 |
| rodEBLN-2 | Rat | Bornavirus | N | 9 | 345 | 33% | 2.6 |
| mlEELN-2 | Microbat | Ebola/Marburgvirus | NP | 11 | 419 | 44% | 2.6 |
| rodEBLN-4 | Rat | Bornavirus | N | 4 | 149 | 45% | 2.7 |
| laEBLN-6 | Elephant | Bornavirus | N | 4 | 137 | 30% | 2.9 |
| meEELN-12 | Wallaby | Ebola/Marburgvirus | NP | 8 | 262 | 39% | 3.1 |
| meEELN-9 | Wallaby | Ebola/Marburgvirus | NP | 9 | 289 | 30% | 3.1 |
| mlEBLL-1E | Microbat | Bornavirus | L | 11 | 339 | 45% | 3.2 |
| meEELN-6 | Wallaby | Ebola/Marburgvirus | NP | 8 | 237 | 33% | 3.4 |
| laEBLN-1 | Elephant | Bornavirus | N | 7 | 189 | 43% | 3.7 |
| meEBLL-2 | Wallaby | Bornavirus | L | 7 | 171 | 33% | 4.1 |
| meEBLL-3 | Wallaby | Bornavirus | L | 7 | 171 | 35% | 4.1 |
| mlEBLL-3 | Microbat | Bornavirus | L | 6 | 145 | 29% | 4.1 |
| meEELN-8 | Wallaby | Ebola/Marburgvirus | NP | 14 | 300 | 27% | 4.7 |

* Individual integrations are listed in Supplementary Tables S1-S7. Multiple BLAST alignments per gene are combined together.
