# Supplementary material for: Unexpected Inheritance: Multiple Integrations of Ancient Bornavirus and Ebolavirus/Marburgvirus Sequences in Vertebrate Genomes
Source: PLoS Pathog. 2010 Jul 29;6(7):e1001030. doi: 10.1371/journal.ppat.1001030 (PMC2912400; doi:10.1371/journal.ppat.1001030)
Supplement: Table S9 — List of species and assemblies analyzed (0.07 MB DOC) [file ppat.1001030.s009.doc]

**Table S9.** ***List of species and assemblies analyzed.***

| Specie | Assembly | Website | Coverage |
| --- | --- | --- | --- |
| Human | hg19 | UCSC genome | complete |
| Chimp | panTro2 | UCSC | 6x |
| Gorilla | gorGor1 | Ensembl 56 | 2x |
| Orangutan | ponAbe2 | UCSC | 6x |
| Macaque | rheMac2 | UCSC | 5x |
| Baboon | Pham20081120 | Baylor College of Medicine | 5.3x |
| Tarsier | tarSyr1 | Broad | 1.82x |
| Marmoset | calJac1 | UCSC | 6x |
| Bushbaby | otoGar1 | UCSC | 1.5x |
| Grey Mouse Lemur | micMur1_draft2 | Broad | 1.93x |
| Mouse | mm9 | UCSC | complete |
| Rat | rn4 | UCSC | 90% |
| Kangaroo Rat | dipOrd1 | Ensembl 56 | 1.85x |
| Squirrel | speTri1_draft1 | Broad | 1.9x |
| Guinea Pig | cavPor3 | UCSC | 6.79x |
| Pika | ochPri2_draft2 | Broad | 1.93x |
| Rabbit | oryCun1 | UCSC | 2x |
| Treeshrew | tupBel1 | UCSC | 2x |
| Alpaca | "vicPac1" | Broad | 2.51x |
| Cow | bosTau4 | UCSC | 7x |
| Dolphin | turTru1 | Broad | 2.59x |
| Pig | sscofa9 | Ensembl 56 | 4x |
| Cat | felCat3 | UCSC | 1.87x |
| Dog | canFam2 | UCSC | 7.5x |
| Panda | Jan 2010 | http://panda.genomics.org.cn | 39-73X |
| Microbat | microbat1 | Ensembl 56 | 1.7x |
| Megabat | pteVam1 | Ensembl 56 | 2.63x |
| Horse | equCab2 | UCSC | 6.79x |
| Hedgehog | eriEur1 | UCSC | 1.86x |
| Shrew | sorAra1 | UCSC | 1.9x |
| Elephant | loxAfr1 | UCSC | 2x |
| Armadillo | dasNov1 | UCSC | 2x |
| Sloth | choHof1 | Ensembl 56 | 2.05x |
| Tenrec | echTel1 | UCSC | 2x |
| Zebrafinch | teaGut1 | UCSC | 6x |
| Chicken | galGal3 | UCSC | 7.1x |
| Lizard | anoGar1 | UCSC | 6.3x |
| Frog | xenTro2 | UCSC | 7.65x |
| Opossum | monDom5 | UCSC | 7.33x |
| Wallaby | Meug20071125 | Baylor College of Medicine | 5.9x |
| Platypus | ornAna1 | UCSC | 6x |
| Medaka | oryLat2 | UCSC | 6.7x |
| Tetraodon | tetNig1 | UCSC | 8.3x |
| Stickleback | gasAcu1 | UCSC | 11x |
| Fugu | fr2 | UCSC | 8.7x |
| Zebrafish | danRer6 | UCSC | 6.5-7X |
| Lamprey | petMar1 | UCSC | 5.9x |
| Elephant Shark | "calMil2007" | http://esharkgenome.imcb.a-star.edu.sg | 1.4x |
| Lancelet | braFlo1 | UCSC | 8.1x |
