# Supplementary figures and images for: Unexpected Inheritance: Multiple Integrations of Ancient Bornavirus and Ebolavirus/Marburgvirus Sequences in Vertebrate Genomes
Source: PLoS Pathog. 2010 Jul 29;6(7):e1001030. doi: 10.1371/journal.ppat.1001030 (PMC2912400; doi:10.1371/journal.ppat.1001030)

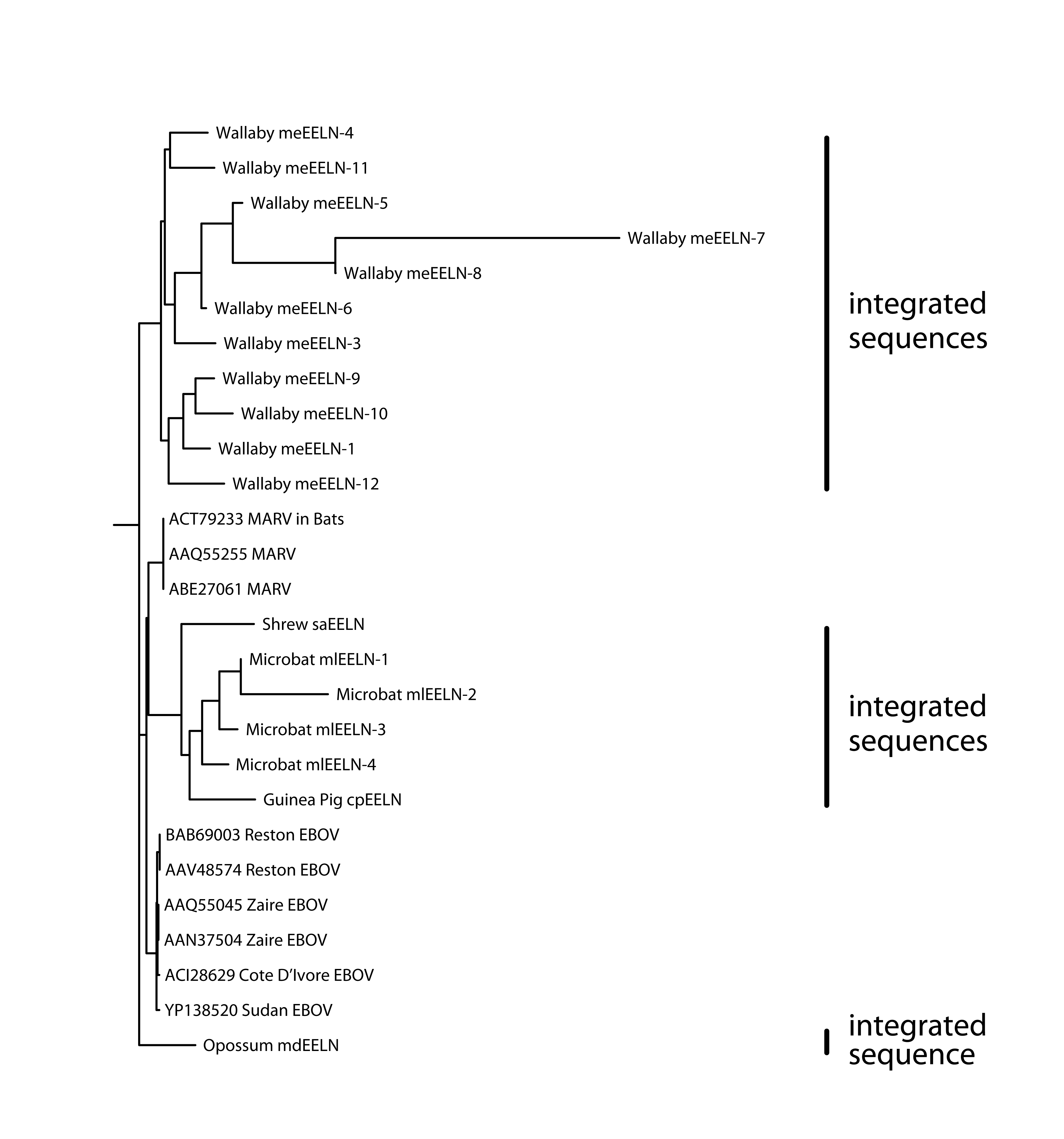

Supplement: Figure S1 — Phylogeny of Filovirus-like NP gene integrations (0.51 MB TIF) [file ppat.1001030.s010.tif]

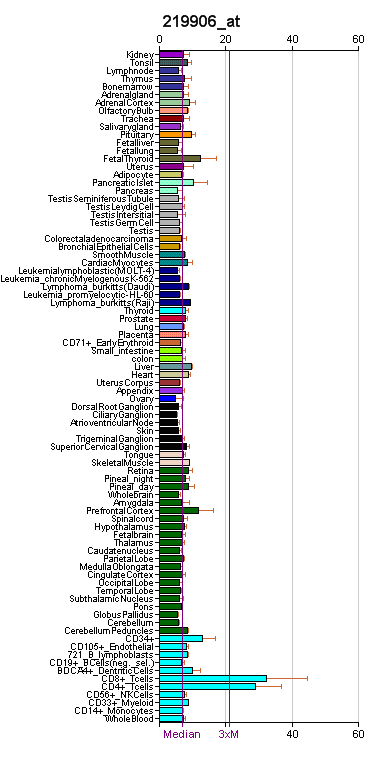

Supplement: Figure S2 — Expression data for the probe 2199906 at that maps onto hsEBLN-2 integration of Borna-like p40 gene in humans [46], [47] (0.06 MB TIF) [file ppat.1001030.s011.tif]

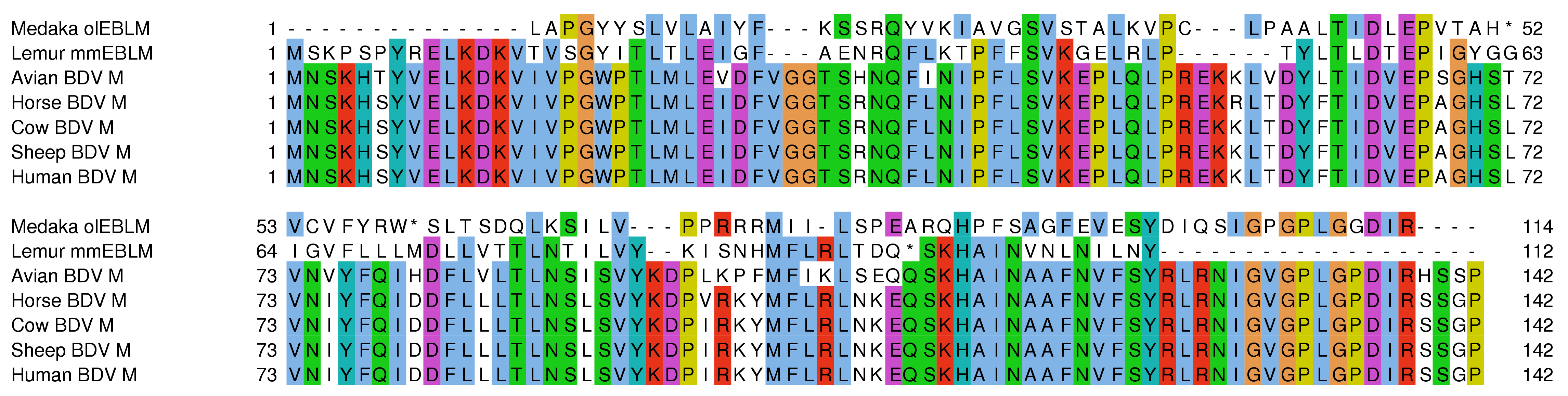

Supplement: Figure S3 — Alignments of Bornavirus matrix proteins and related endogenous sequences. The indicated endogenous sequences are compared with sequences of Bornavirus isolated from a variety of species including: horse (AJ311524), cow (AB246670), sheep (AY066023), human (AB032031). We used the default color scheme for Clustal W alignment in the Jalview program. (1.08 MB TIF) [file ppat.1001030.s012.tif]
